# Development of a novel definitive scoring system for an enteral feed-only model of necrotizing enterocolitis in piglets

**DOI:** 10.3389/fped.2023.1126552

**Published:** 2023-04-17

**Authors:** Mecklin V. Ragan, Samantha J. Wala, Nitin Sajankila, Audrey F. Duff, Yijie Wang, Samuel G. Volpe, Ameer Al-Hadidi, Zachary Dumbauld, Nanditha Purayil, Joseph Wickham, Miriam R. Conces, Belgacem Mihi, Steven D. Goodman, Michael T. Bailey, Gail E. Besner

**Affiliations:** ^1^ Center for Perinatal Research, Nationwide Children's Hospital, Columbus, OH, United States; ^2^ Department of Pediatric Surgery, Nationwide Children’s Hospital, Columbus, OH, United States; ^3^ Center for Microbial Pathogenesis, Nationwide Children’s Hospital, Columbus, OH, United States; ^4^ Department of Pathology, Nationwide Children’s Hospital, Columbus, OH, United States

**Keywords:** necrotizing enterocolitis (NEC), piglet, enteral feed, microbiome, colostrum

## Abstract

**Introduction:**

Necrotizing enterocolitis (NEC) is a complex inflammatory disorder of the human intestine that most often occurs in premature newborns. Animal models of NEC typically use mice or rats; however, pigs have emerged as a viable alternative given their similar size, intestinal development, and physiology compared to humans. While most piglet NEC models initially administer total parenteral nutrition prior to enteral feeds, here we describe an enteral-feed only piglet model of NEC that recapitulates the microbiome abnormalities present in neonates that develop NEC and introduce a novel multifactorial definitive NEC (D-NEC) scoring system to assess disease severity.

**Methods:**

Premature piglets were delivered *via* Caesarean section. Piglets in the colostrum-fed group received bovine colostrum feeds only throughout the experiment. Piglets in the formula-fed group received colostrum for the first 24 h of life, followed by Neocate Junior to induce intestinal injury. The presence of at least 3 of the following 4 criteria were required to diagnose D-NEC: (1) gross injury score ≥4 of 6; (2) histologic injury score ≥3 of 5; (3) a newly developed clinical sickness score ≥5 of 8 within the last 12 h of life; and (4) bacterial translocation to ≥2 internal organs. Quantitative reverse transcription polymerase chain reaction was performed to confirm intestinal inflammation in the small intestine and colon. 16S rRNA sequencing was performed to evaluate the intestinal microbiome.

**Results:**

Compared to the colostrum-fed group, the formula-fed group had lower survival, higher clinical sickness scores, and more severe gross and histologic intestinal injury. There was significantly increased bacterial translocation, D-NEC, and expression of *IL-1α* and *IL-10* in the colon of formula-fed compared to colostrum-fed piglets. Intestinal microbiome analysis of piglets with D-NEC demonstrated lower microbial diversity and increased Gammaproteobacteria and Enterobacteriaceae.

**Conclusions:**

We have developed a clinical sickness score and a new multifactorial D-NEC scoring system to accurately evaluate an enteral feed-only piglet model of NEC. Piglets with D-NEC had microbiome changes consistent with those seen in preterm infants with NEC. This model can be used to test future novel therapies to treat and prevent this devastating disease.

## 1. Introduction

Necrotizing enterocolitis (NEC) is the leading cause of death due to gastrointestinal disease in premature infants, with a mortality rate as high as 20%–30% for infants requiring surgery (1–3). As described by Bell et al*.* (4), NEC classically presents in a susceptible preterm infant after the initiation of enteral feeds, with rapid onset of abdominal distension, feed intolerance, bloody diarrhea, and intestinal necrosis in the most severe cases (5, 6). Diagnosis is based on a multifactorial modified Bell's staging criteria, which examines systemic, abdominal, and radiographic manifestations (4). Currently, it is widely accepted that the pathophysiology underlining NEC involves gut immaturity, exaggerated inflammatory response, and bacterial translocation across the intestinal barrier (5). In addition, it has been shown that preterm infants with NEC have intestinal dysbiosis, with an overall increase in Proteobacteria, particularly Enterobacteriaceae, and a decrease in Firmicutes and Bacteroidetes (7). This has been further described in premature piglets with NEC by Touloukian et al*.* who demonstrated that the disease is associated with ileal dysbiosis, characterized by the overrepresentation of *Clostridium* species, and members of the Actinobacteria and Cyanobacteria phyla (8). This imbalance in the intestinal microbiota results in blooms of opportunistic pathogens that are known to stimulate toll-like receptor 4 (TLR4) signaling, which is a significant player in the development of this disease (9). Several studies have demonstrated that breast milk plays an important role in preventing the development of NEC through the maintenance of epithelial barrier homeostasis *via* growth factors, enhancing the colonization of beneficial commensal bacteria in the intestine, and by preventing the expansion of Enterobacteriaceae *via* maternal immunoglobulin A (IgA) (10, 11). Despite the significant progress made in our understanding of the pathophysiology of NEC, existing therapeutic strategies remain elusive, leaving clinicians unarmed against this devastating disease (2, 10, 11). It is clear that a better understanding of the disease is required for the development of definitive diagnostics and novel efficacious treatments. Hence, several animal models have been developed, with rodents being most commonly used to study NEC. Rodent models often depend on repeated exposures to different stresses to induce NEC, including administration of the Gram-negative bacterial cell wall product lipopolysaccharide (LPS), exposure to hypoxia, exposure to hypothermia, and the administration of formula feeds (12, 13). The use of rodents has multiple advantages, including their small size, ease of maintenance, short gestation, and the abundance of genetically modified animals which has allowed a substantial advancement in the comprehension of NEC pathophysiology. On the other hand, rodents display significant size, developmental and physiologic differences compared to humans, thus limiting the relevance of their use as models to study NEC (14). Therefore, premature piglets have emerged as an alternative to rodent models of NEC. In addition to their body weight being comparable to a preterm human neonate, the piglet intestine more closely resembles the human intestine histologically and physiologically (12, 13, 15, 16). Delivery of preterm piglets at 90% term gestation provides a natural brief period of hypoxia and hypothermia, similar to human deliveries (15, 17). However, unlike rodent models where the activation of TLR4 combined with hypoxic and hypothermic stresses are required for the induction of NEC, premature piglets develop NEC spontaneously upon initiation of enteral feeds, as do human premature infants (12, 15, 16). This was confirmed by Bjornvad et al*.* in 2008 when they characterized the intestinal changes associated with formula feeds compared to colostrum in preterm piglets (18). Formula-fed piglets had increased mucosal inflammation and loss of mucosal integrity, as is observed in premature neonates with NEC (18). Pigs are also known to have a comparable microbiome to that of humans (19–23) allowing for a more detailed analysis of changes in the microbiome with NEC. In addition, confirmation of results obtained in rodents using a large animal model of disease can be very useful in obtaining Food and Drug Administration (FDA) approval for the testing of novel therapeutic agents in humans.

Several groups have demonstrated that premature piglet models of NEC have reproducible and characteristic clinical changes and intestinal injury similar to that seen in human NEC (8, 24–26). While some groups rely solely on macroscopic damage to assess NEC severity (24, 27, 28), others have quantified the cellular and architectural changes that occur during NEC in piglets, adapting similar criteria to that used in the rat NEC model (24, 27, 28). Interestingly, a microbial analysis revealed that NEC in premature piglets is associated with ileal dysbiosis, characterized by the overrepresentation of *Clostridium* species, and members of the Actinobacteria and Cyanobacteria phyla (8).

Although piglets have been used to study NEC since 1972, there is still no consensus on the definition of the disease in this model (8), making it difficult to compare the results between different research groups. In addition, current pig NEC models typically utilize total parenteral nutrition (TPN) for the first 48 h of life, followed by enteral feeds (29–31). Here, we present a multifactorial definitive NEC scoring system using a simplified enteral feed-only piglet model of the disease to standardize the global injury that occurs.

## 2. Materials and methods

### 2.1. Cesarean delivery of premature piglets

All animal studies were carried out in accordance with the guidelines of the Institutional Animal Care and Use Committee (IACUC) of the Research Institute at Nationwide Children's Hospital (protocol #AR18-00062). White Yorkshire × Landrace sows were purchased from Oak Hill Genetics (Ewing, IL). Sows were acclimatized to our facility for five days before terminal cesarean section delivery, performed on gestational day E104, with full term gestation being E114. Piglets delivered at E104 are roughly equivalent to premature infants born at 32 weeks gestation (16).

Sows were sedated with an intramuscular injection of telazol (0.4–1.0 mg/kg), ketamine (1–2.5 mg/kg), and xylazine (1–2.5 mg/kg), followed by an intramuscular injection of glycopyrrolate (0.01 mg/kg). After endotracheal intubation, anesthesia was maintained throughout the cesarean section using 1%–4% isoflurane. After induction of adequate anesthesia, laparotomy was performed through a lower midline incision to expose the uterus and deliver the piglets. The umbilical cord of each piglet was milked to ensure adequate placental blood transfusion prior to cord ligation. The time from induction of anesthesia to piglet delivery was kept at a minimum to avoid excess exposure of the premature piglets to anesthesia. After all piglets were delivered (typically within 10–15 min after skin incision), the sow was euthanized with IV or intracardiac Euthasol® (1 ml/4.5 kg) and thoracotomy, and the laparotomy closed.

### 2.2. Piglet resuscitation

Piglets were immediately dried and resuscitated after birth by clearing the remaining mucous from the airways using suction and providing positive pressure bag-mask ventilation as needed. Once piglets showed signs of independent breathing, 1 ml of iron dextran (Vedco, Saint Joseph, MO) was administered intramuscularly to prevent iron deficiency anemia, one drop of sublingual Doxapram (WEST-WARD, Eatontown, NJ) was administered to stimulate respiration, and sublingual glucose paste (Insta-Glucose, Valeant Pharmaceuticals, Bridgewater, NJ) was provided to prevent hypoglycemia. Doxapram was re-dosed as needed to piglets that were slow to breathe independently and require additional stimulation. Once resuscitated, piglets were placed in temperature- and oxygen-controlled small animal intensive care units (Suburban Surgical Co, Wheeling, IL) to maintain a temperature of 35.5°C–39.5°C with FiO2 of 40%. Once stabilized, typically within 60 min, each piglet was weighed and sexed. A 6-French transbuccal orogastric feeding tube (Cardinal Health, Dublin, OH) was introduced into the stomach through a small puncture made with the needle in the cheek. 3-0 polypropylene sutures were used to secure the feeding tube to the cheek, snout, and forehead, with additional securement of the tubes using Elastoplast tape. After tube placement was confirmed by auscultation, piglets received 10 ml of dextrose (25 g) suspended in 500 ml Pedialyte (Abbott, Columbus, OH). Piglets from 4 separate experiments were randomized regardless of sex into two different experimental groups—the colostrum-fed group (total *n* = 17) and the formula-fed group (total *n* = 18) (Supplementary Figure S1).

### 2.3. Induction of NEC

Piglets were fed every 3–4 h for a total of 6–8 feeds/day. The initial volume goal of colostrum or formula feeds was 240 ml/kg/day and was decreased to 120 ml/kg/day in later experiments to avoid potential over-hydration. Oral glucose paste was administered if there was concern for hypoglycemia. Feeding volumes were scaled up over the first two days. 60% of goal volume was administered as colostrum or formula on day 1, with the remaining 40% of the goal volume achieved with Pedialyte supplementation. On day 2, 75% of goal volume was administered as colostrum or formula, with the remaining 25% of the goal volume achieved with Pedialyte. On days 3 to 5, the complete goal volume was administered as colostrum or formula.

Fresh frozen bovine colostrum (BC) was obtained from OSU Waterman Farms (Columbus, OH). The colostrum provided was medium grade in quality, between 20 and 50 mg/ml of immunoglobulins, as assessed on the farm by density measurement using a Brix Refractometer. The colostrum was diluted to a 50% concentration with distilled autoclaved water upon thawing since higher concentrations led to gastric bezoar formation. Piglets in the colostrum-fed group received colostrum for the entire experiment. In contrast, piglets in the formula-fed group received fresh BC for 24 h, followed by Neocate Jr (1.0 kcal/ml; 120 kcal/kg/day) (Nutricia, Zoetermeer, Netherlands) for the remainder of the experiment to induce intestinal injury. All milk products were prepared daily and stored in a 4°C refrigerator. Aliquots were taken before each feed and warmed for 20–30 min prior to administration.

### 2.4. Quantification of IgG in bovine colostrum

IgG is the major immunoglobulin present in BC (32, 33), and its use as a marker for BC quality is well-documented in the literature (32–36). To further assess colostrum quality, samples were thawed and centrifuged four times at 10,000 g for 20 min each at 4°C to remove cells and fat. BC IgG was quantified using a bovine IgG ELISA kit (Bethyl Laboratories Inc., Montgomery, TX) following the manufacturer's instructions with minor modifications. Samples were diluted to 1:500,000 and plated in triplicate on 96-well plates using 3,3′,5,5′tetramethylbenzidine (TMB) as substrate. Absorbance OD_450 nm_ was measured using a Spectramax M2 microplate reader equipped with SoftMax Pro 5.4 software (Molecular Devices, San Jose, CA). IgG concentrations were calculated using standard curves.

### 2.5. Clinical sickness scores (CSS)

Piglets were monitored continuously throughout the experiment. Although unblinded to groups, we noted observable differences in the piglets in the two different groups. Thus, we initiated a new scoring system, the clinical sickness score (CSS), to define our observations. CSS were assigned at the time of each feed, beginning at 12 h of life. The CSS was comprised of 4 different categories: motor/tone, verbal, alertness, and body color, with scores ranging from 0 to 2 per category depending on severity, for a maximal total CSS of 8 (Table 1). This was reported as an average CSS for all piglets in each group at each time point. If a piglet was euthanized prior to the end of the experiment, that piglet was given a score of an 8 for all remaining time points of the experiment.

**Table 1:** Clinical sickness score (CSS). CSS was documented at the time of each feed (every 3–4 h) beginning at 12 h of life. Each piglet received a score of 0–2 in the four different categories (motor/tone, verbal, alertness, and body color) for a total CSS of 0–8.

Score	Motor/Tone	Verbal	Alertness	Body Color
0	Ambulating or standing or good tone (able to keep head up)	Vocal	Awake and alert	Pink
1	Recumbent, good tone (can keep head up, tongue not sticking out)	Intermittently vocal or vocal with stimulation	Responsive to stimuli (being held, feeding, back scratch)	Pale or dark distal extremities (fingers/toes), color change to snout
2	Recumbent, poor tone (cannot keep head up, tongue sticking out)	Non-vocal	Unresponsive to painful stimuli (example, pinching, rectal temperature)	Gray

### 2.6. Euthanasia and necropsy

In alignment with our IACUC protocol, piglets were euthanized if they lost more than 25% of their birth weight, had a single temperature >40.5°C, or were noted to have extreme lethargy. Additionally, piglets were euthanized if two of the following were observed during two consecutive feeds: loss of >20% of birth weight, significant lethargy/decreased activity, temperature >40°C, bloody diarrhea, abdominal distension, emesis, or rapid, labored, or shallow breathing. Euthanasia was performed using an intramuscular injection of ketamine (0.3–0.6 ml/kg) and xylazine (0.1–0.2 ml/kg), followed by an intraperitoneal injection of Euthasol® (1–2 ml/kg).

### 2.7. Gross injury scoring

**Figure 1:**
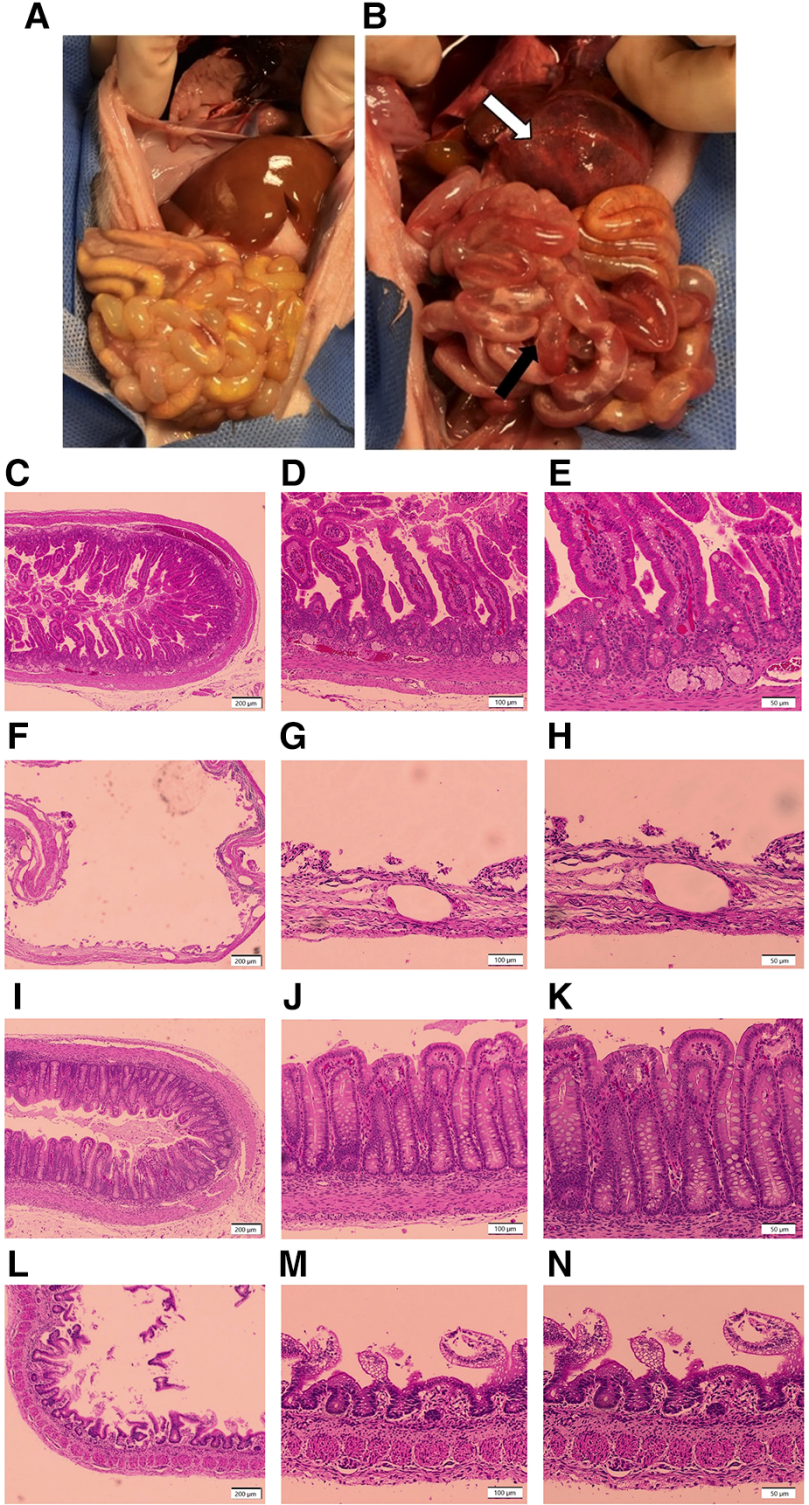
Representative gross and histologic intestinal images. (**A,B**) Gross Injury. (**A**) Image from a healthy piglet from the colostrum-fed group with no intestinal injury, corresponding to a gross injury score of 1; (**B**) image from a piglet in the formula-fed group, with extensive hemorrhage of the stomach (white arrow), and hyperemia and edema with focal areas of pneumatosis intestinalis in the mid small bowel (black arrow), corresponding to a gross injury score of a 5. (**C–N**) Histologic Injury. (**C–E**) Image of healthy small intestine from a piglet in the colostrum-fed group with no evidence of damage, corresponding to a histologic injury score of 1 at (**C**) 4×, (**D**) 10×, and (**E**) 20× magnification; (**F–H**) image of small intestine from a piglet in the formula-fed group demonstrating transmural necrosis, scant villi, and widespread pneumatosis, corresponding to a histologic injury score of 5 at (**F**) 4×, (**G**) 10×, and (**H**) 20× magnification; (**I–K**) image of healthy colon from a piglet in the colostrum-fed group with no evidence of damage, corresponding to a histologic injury score of 1 at (**I**) 4×, (**J**) 10×, and (**K**) 20× magnification; (**L–N**) image of colon from a piglet in the formula-fed group demonstrating mucosal sloughing with red blood cell infiltration, corresponding to a histologic injury score of 4 at (**L**) 4×, (**M**) 10×, and (**N**) 20× magnification.

After euthanasia, a midline laparotomy was performed, and the intestine examined *in situ*. A gross injury score was assigned to the small intestine (proximal, mid, and distal segments) and colon based on the most severely affected area assessed independently by two graders using an adaptation of a published scoring system (16, 37). Scores ranged from 1 (no injury) to 6 (most severe injury) using the criteria shown in Table 2. The highest overall score assigned to any segment was used to assign the animal to either no gross injury (grades 1–3) or gross injury (grades 4–6) (Figures 1A,B) (16, 37).

**Table 2:** Gross injury scoring system. Gross injury scores were assigned to the proximal small intestine, mid small intestine, distal small intestine, and colon of each animal. Scores of 1–3 were considered to have no gross injury, and scores of 4–6 were considered to have gross injury. The final score assigned to an animal was the highest score in any given section.

Score	Macroscopic Findings	Injury Severity
1	Absence of Injury	No Gross Injury
2	Hyperemia in a small section of intestine	
3	Hyperemia throughout the intestine, edema throughout the intestine, and hemorrhage in a small section of intestine	
4	Hemorrhage throughout the intestine	Gross Injury
5	Necrosis in a small section of intestine and pneumatosis intestinalis	
6	Transmural necrosis throughout the intestine, pneumatosis intestinalis, and intestinal perforation	

### 2.8. Histologic injury scoring

Tissue was collected from the small intestine (proximal, mid, and distal) and colon, fixed in 10% formalin (Fisher Scientific, Pittsburgh, PA) for 24 h, followed by 70% ethanol (Fisher Scientific, Waltham, MA). Samples were paraffin-embedded and stained with hematoxylin and eosin (H&E). Histologic sections were reviewed by light microscopy by at least two independent graders, including a board-certified pediatric pathologist, in a blinded fashion. Histologic injury scores were assigned based on a previously published grading system (28). Scores ranged from 1 (no damage) to 5 (transmural necrosis) (Table 3). The final score assigned to any animal was the highest score identified in any intestinal segment, with scores of 3 or above consistent with NEC. Scores were categorized as no histologic injury (grades 1–2), moderate histologic injury (grades 3–4), or severe histologic injury (grade 5) (Figures 1C–N). Images were taken using an Olympus U-Tv0.5XC-3 microscope (Tokyo, Japan).

**Table 3:** Histologic injury scoring system. Histologic injury scores were assigned to the proximal small intestine, mid small intestine, distal small intestine, and colon of each animal. The final score assigned to an animal was the highest score in any given section. Scores of 1–2 were considered no histologic injury, scores of 3–4 were considered moderate histologic injury, and a score of 5 was considered severe histologic injury.

Small Intestine Histologic NEC Score		Colon Histologic NEC Score		Injury Severity
Score	Microscopic Findings	Score	Microscopic Findings	
1	No damage	1	No damage	No Histologic Injury
2	Epithelial cell lifting, majority of villi intact	2	Minimal mucosal breakage	
3	Necrosis of epithelial cells to mid-villus level, blunting of villi	3	Mucosal sloughing with RBC infiltration	Moderate Histologic Injury
4	Necrosis of entire villi, occasional villi, pneumatosis	4	Pneumatosis with incomplete mucosal necrosis, OR complete mucosal necrosis without pneumatosis	
5	Transmural necrosis, scant villi, widespread pneumatosis	5	Pneumatosis with transmural necrosis	Severe Histologic Injury

### 2.9. Bacterial translocation

Liver, spleen, and mesenteric lymph nodes were collected in a sterile fashion at the time of necropsy, snap frozen, and stored at −80°C until use. Samples were thawed on ice, weighed, and homogenized in 2 ml of PBS before plating 50 µl of the homogenized mixture on brain heart infusion (BHI) plates for overnight, aerobic incubation at 37°C. BHI is a general-purpose growth medium for culturing non-fastidious Gram-positive and Gram-negative bacteria (38). The number of colony-forming units (CFUs) for each organ was recorded after 24 h of growth and standardized per gram of tissue.

### 2.10. Definitive NEC (D-NEC) scoring

Piglets were determined to have D-NEC if they met at least 3 of the following four criteria: Gross injury score ≥4 of a maximum of 6, histologic injury score ≥3 of a maximum of 5, CSS ≥5 of a maximum of 8 in the last 12 h of life, and bacterial translocation of ≥1 CFU/g of tissue to two or more internal organs (liver, spleen, mesenteric lymph nodes).

### 2.11. RNA isolation, cDNA synthesis, and quantitative real-time PCR (qRT-PCR)

Intestinal specimens of proximal, mid, and distal small bowel and colon were collected during necropsy and stored at −80°C until use. Total RNA was isolated using the Purelink RNA mini kit (Thermo Fisher, Waltham, MA) according to the manufacturer's instructions. Samples were thawed on ice and weighed. Tissues (0.5–0.9 g) were homogenized in lysis buffer with 1.0 mm Zirconia beads (Biospec Products, Bartlesville, OK) in a TissueLyser II (Qiagen, Germantown, MD) at 30 Hz for 1 min, repeated six times. cDNA was synthesized using the Superscript IV VILO cDNA synthesis kit with ezDNAse Enzyme to remove genomic DNA (Thermo Fisher, Waltham, MA). Quantitative real-time PCR (qRT-PCR) was performed using primers for interleukin-1*α* (*IL-1α*) and *IL-10*, and PowerUp SYBR Green PCR Master mix (Thermo Fisher, Waltham, MA) using a QuantStudio™ 3 System (ThermoFisher, Waltham, MA) (Table 4) (39–41). Hypoxanthine-guanine phosphoribosyltransferase (*HPRT*) (Integrated DNA Technologies, Coralville, IA) was used as an endogenous control The cycling program consisted of a 2-minute hold stage at 50°C, a 10-minute hold stage at 95°C, followed by 40 cycles of 15 s at 95°C followed by 1-min at 60°C. Target gene expression was analyzed using the 2^−ΔΔCt^ method.

**Table 4:** Primer gene sequences.

Primer	Sequence	
*IL-1α* (39)	Forward	CAGCCAACGGAAGATTCTG
	Reverse	ATGGCTTCCAGGTCGTCAT
*IL-10* (40)	Forward	GGAGAAGCTGAAGACCCTCA
	Reverse	CGGCCTTGCTCTTGTTTTCA
*HPRT* (41)	Forward	GGACTTGAATCATGTTTGTG
	Reverse	CAGATGTTTCCAAACTCAAC

### 2.12. DNA extraction and 16S rRNA gene sequencing

Intestinal contents were aseptically collected from the colon of all piglets at sacrifice and frozen at −80°C until use. Approximately 100 mg of colonic content was subjected to DNA extraction using the QIAamp DNA Mini Kit (Qiagen, Hilden, Germany) per manufacturer's instructions with slight modifications. Contents were incubated for 45 min at 37°C in lysozyme-mutanolysin buffer (pH 8.0) containing 22 mg/ml lysozyme, 0.1 U/ml mutanolysin, 20 mM TrisHCL, 1.2% Triton-x (Sigma Aldrich, St. Louis, MO), and 2 mM EDTA (Thermo Fisher Scientific, Waltham, MA), followed by homogenization for 150 s with 0.1 mm zirconia beads. Samples were incubated at 95°C for 5 min with InhibitEx Buffer, then incubated at 70°C for 10 min with Proteinase K and Buffer AL (Qiagen). Following this step, the QIAamp DNA Mini Kit isolation protocol was followed beginning with the ethanol step. DNA was quantified with the Qubit 2.0 Fluorometer (Life Technologies, Carlsbad, CA) using the dsDNA Broad Range Assay Kit and stored at −20°C prior to 16S rRNA gene sequencing.

Colonic content DNA samples were submitted to the Genomic Services Core at the Institute for Genomic Medicine at Nationwide Children's Hospital (Columbus, OH) for library preparation and high-throughput sequencing. Paired-end (300 nt forward and reverse) sequences of the V4 hypervariable region of the 16S rRNA gene (515F-806R) were generated by Illumina MiSeq. Quantitative Insights Into Microbial Ecology (QIIME) 2.0 (42) and DADA2 (43) were utilized for downstream amplicon processing, quality control, diversity analyses, and taxonomic assignment using a trained classifier built from the ribosomal RNA database SILVAv138 (44, 45). For quality control, sequences were truncated from the 3′ end to 280 nt (forward reads) or 250 nt (reverse reads) to achieve a quality score of at least 20, and the first 20 nt were trimmed from the 5′ end of both forward and reverse reads as these early positions may contain errors. Sequences that did not meet these criteria were discarded. After quality control, taxa denoted as eukaryotic or “unassigned” were also removed. Subsequent diversity analyses were carried out at a sequencing depth of 18,755 sequences per sample. Evenness, richness (number of observed species), and Shannon's Diversity Index (46) were calculated to measure alpha diversity within samples on the basis of feeding regimen and occurrence of NEC. The weighted UniFrac distance matrix (47) was applied to measure beta diversity, and the EMPeror software package (48) was used to construct three-dimensional principal coordinate (PCoA) plots to visualize differences based on feeding regimen and occurrence of NEC. Relative abundance was calculated for the class, family, and genus taxonomic levels on an individual sample basis as well as abundances within feeding regimen and occurrence of NEC.

### 2.13. Statistical analyses

All statistical analyses were performed using GraphPad Prism, Version 9 (GraphPad Software, Inc. La Jolla, CA). Nonparametric analysis between groups was performed using the Mann–Whitney *U*-test. Survival data were analyzed using a log-rank (Mantel-Cox) test.

Differences in bacterial community composition were assessed by comparing alpha and beta diversity between feeding regimens (i.e., colostrum fed vs. formula fed) and between pigs that developed NEC vs. those with No NEC collapsed across the two feeding regimens. Evenness, richness, and Shannon Diversity Index alpha diversity metrics used the Kruskal-Wallis test for pairwise comparisons. Differences in beta diversity weighted UniFrac distances were analyzed by permutational multivariate analysis of variance (PERMANOVA) with 999 randomizations of the data. Relative abundance data were analyzed using the Kruskal-Wallis test for pairwise comparisons, and taxa that were present in fewer than 10% of samples (present in fewer than 3/26 samples) were omitted from abundance analyses. The statistical alpha level was set to 0.05 for all analyses.

## 3. Results

### 3.1. Bovine colostrum feeding prevents the early death of premature piglets

In preliminary experiments, piglets that were exclusively fed with commercial human formulas were found to have a rapid deterioration of their health with early death within the first 24 h of life. We investigated the use of Sow Colostrum Replacer (APS LaBelle, Phoenix, AZ), but this had a minor effect on preventing early death. Unlike infants, piglets do not undergo maternal-placental transfer of immunoglobulins and instead depend almost exclusively on postnatal transfer of maternal IgG *via* colostrum (13, 49–52). We ultimately chose bovine colostrum because of its high levels of beneficial immunoglobulins to help protect against early animal mortality, and human breast milk or porcine colostrum were not available in the volumes needed. Piglets subsequently received BC for 24 h prior to the introduction of formula feeding to induce NEC. This led to a significant improvement in early mortality, however, the effect was variable depending upon the lot of BC used. Based on this, we investigated the IgG content of the different BC lots by ELISA. Consistent with previous reports, we found that the efficacy of the colostrum in preventing early death was dependent upon its IgG content (33). We measured the IgG content in 8 different batches of colostrum from 7 sows. The range of IgG varied widely, from 1.123 mg/ml to 94.898 mg/ml. We found that colostrum with IgG levels greater than 5 mg/ml reduced early death (data not shown). Thus, for all subsequent experiments, we used colostrum that contained a minimum of 5 mg/ml of IgG.

In addition to the use of BC, we found that rapid piglet delivery to decrease anesthesia exposure time, the use of oxygen and temperature-controlled incubators, and the administration of iron dextran to prevent iron deficiency anemia, doxapram to simulate respiration, and sublingual glucose to prevent hypoglycemia significantly improved piglet survival during the first 24 h of life.

### 3.2. Formula feeding adversely affects intestinal health in premature piglets

**Figure 2:**
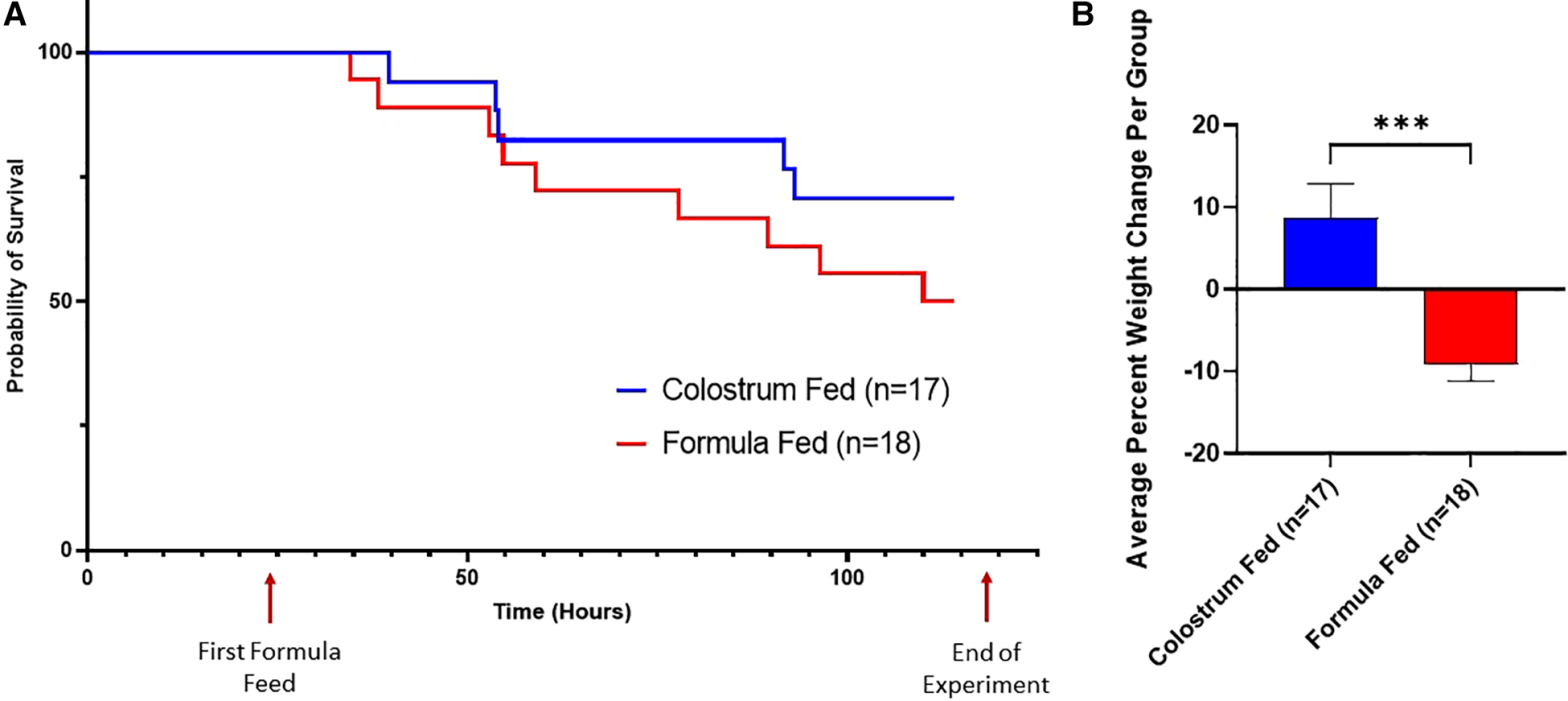
Survival curves and average percent weight change. (**A**) Survival Curves. These survival curves depict all-cause mortality (NEC plus non-NEC deaths) over the course of the experiment. (**B**) Weight Change. The percent weight change per piglet was averaged for each group. Variability of sample means was reported as a standard error of the mean (SEM) and statistical significance level was set to *α *= 0.05. Statistical analysis was carried out using the Mann–Whitney *U*-test (***p = 0.0001).

**Figure 3:**
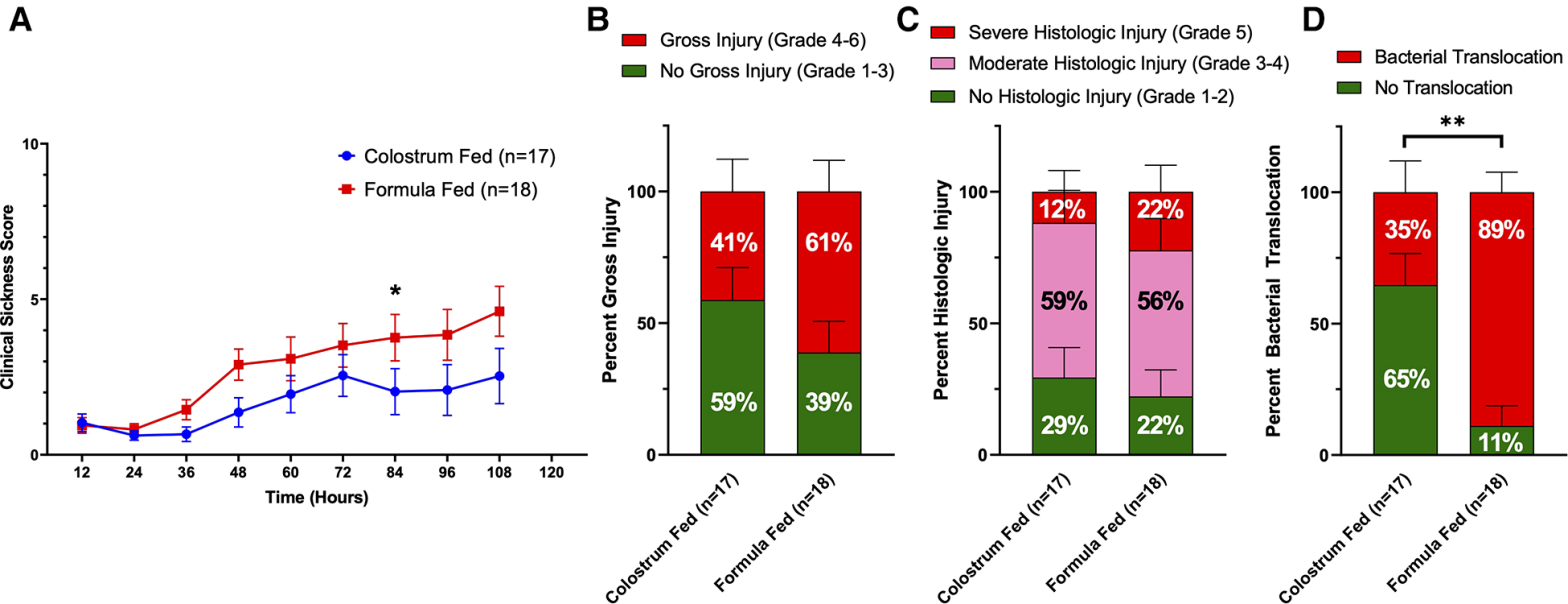
Components of D-NEC score. (**A**) Clinical Sickness Scores (CSS). Each data point represents the average CSS for all piglets in that group. If a piglet was euthanized prior to the end of the experiment, that piglet was given a score of an 8 for all remaining time points of the experiment; (**B**) Gross Injury Scores; (**C**) Histologic Injury Scores; (**D**) Bacterial Translocation. Variability of sample means was reported as a standard error of the mean (SEM) and statistical significance level was set to *α *= 0.05. Statistical analyses for gross injury score, histologic injury score, and bacterial translocation were carried out using Sidak’s multiple comparison test (***p < 0.001). *p* values were calculated at each timepoint for CSS using the Mann–Whitney *U*-test (*p = 0.05).

Piglets in the formula-fed group had increased all-cause mortality compared to piglets in the colostrum-fed group, regardless of sex (Figure 2A). Furthermore, piglets in the formula-fed group lost significantly more weight compared to piglets in the colostrum-fed group (−9.9% vs. +5.3%, *p* = 0.0001) (Figure 2B) and had significantly higher CSS than piglets in the colostrum-fed group (Figure 3A).

Upon necropsy, we found that 61% (11 of 18) of animals in the formula-fed group had gross evidence of intestinal injury consistent with NEC, compared to 41% (7 of 17) of animals in the colostrum-fed group (Figure 3B). The highest degree of gross injury in both groups was seen in the mid-small bowel, with an average gross injury score of 3.3 in the formula-fed group and 2.5 in the colostrum-fed group. The lowest degree of gross injury in both groups was seen in the proximal small bowel, with an average score of 1.9 in the formula-fed group and 1.7 in the colostrum-fed group (Supplementary Figure S2). While these findings were not statistically significant, average gross injury in all sections of bowel was greater in the formula-fed group than the colostrum-fed group.

Histological examination of the intestine revealed that 78% (14 of 18) of animals in the formula-fed group had histologic intestinal injury consistent with NEC, with 56% having moderate NEC and 22% having severe NEC (Figure 3C). 71% (12 of 17) of animals in the colostrum-fed group had histologic injury consistent with NEC, with 59% having moderate NEC and 12% having severe NEC. In both groups, the mid-small bowel was found to have the highest degree of histologic injury, with an average score of 3.2 in the formula-fed group and 2.9 in the colostrum-fed group (Supplementary Figure S2). While these findings were not statistically significant, average histologic injury in all sections of bowel was greater in the formula-fed group than the colostrum-fed group.

Since the intestinal injury that occurs with NEC results in gut barrier failure, we next assessed intestinal barrier function by detecting bacterial translocation to the liver, spleen, and mesenteric lymph nodes. Bacterial translocation was detected in 89% of piglets in the formula-fed group compared to only 35% of piglets in the colostrum-fed group (*p *= 0.0016) (Figure 3D).

### 3.3. Formula feeding increases Definitive NEC (D-NEC), increases death due to NEC, and promotes colonic inflammation in premature piglets

**Figure 4:**
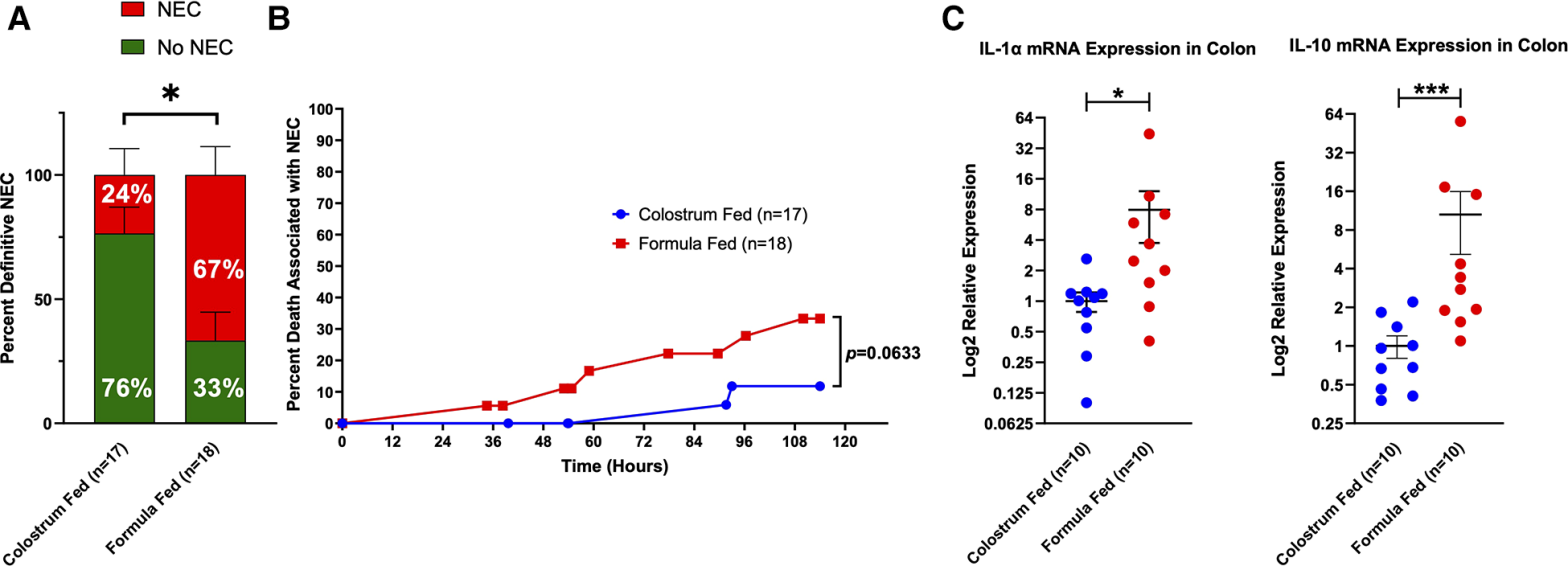
Definitive NEC (D-NEC) incidence, percent death associated with NEC, and colonic inflammation. (**A**) Incidence of D-NEC. Statistical analysis was carried out using the Mann–Whitney *U*-test (*p = 0.0176); (**B**) Percent Death Associated with NEC. Each data point represents death of a piglet that was determined to have D-NEC. Piglets that survived to the end of the experiment and were found to have D-NEC were not included in this graph; (**C**) Colonic Inflammation. Expression levels of *IL-1α* and *IL-10* mRNA in the colon were determined by qRT-PCR. Data were analyzed using the Mann–Whitney *U*-test (*p = 0.0115, ***p = 0.0005). Variability of sample means was reported as a standard error of the mean (SEM) and statistical significance was set to an *α *= 0.05.

D-NEC criteria were met if a piglet met at least 3 of the following 4 criteria: gross injury score of ≥4, histologic injury score of ≥3, CSS of ≥5 in the last 12 h of life, or bacterial translocation to ≥2 internal organs (liver, spleen, mesenteric lymph nodes). 77% (12 of 18) of animals in the formula-fed group had D-NEC compared to 24% (4 of 17) of animals in the colostrum-fed group (*p *= 0.0176) (Figure 4A). This was not affected by the sex of the piglet.

Piglets in the formula-fed group tended to die during the experiment due to D-NEC more frequently than piglets in the colostrum-fed group (33.3% vs. 11.8%) (Figure 4B). qRT-PCR was performed on samples of the mid and distal small bowel and colon from 10 piglets in each group. There was significantly increased mRNA expression for *IL-1α* (*p *= 0.0115) and *IL-10* (*p *= 0.0005) in colon specimens from the formula-fed group compared to the colostrum-fed group (Figure 4C).

### 3.4. Formula feeding and NEC in premature piglets are associated with reduced diversity of the intestinal microbiome

**Figure 5:**
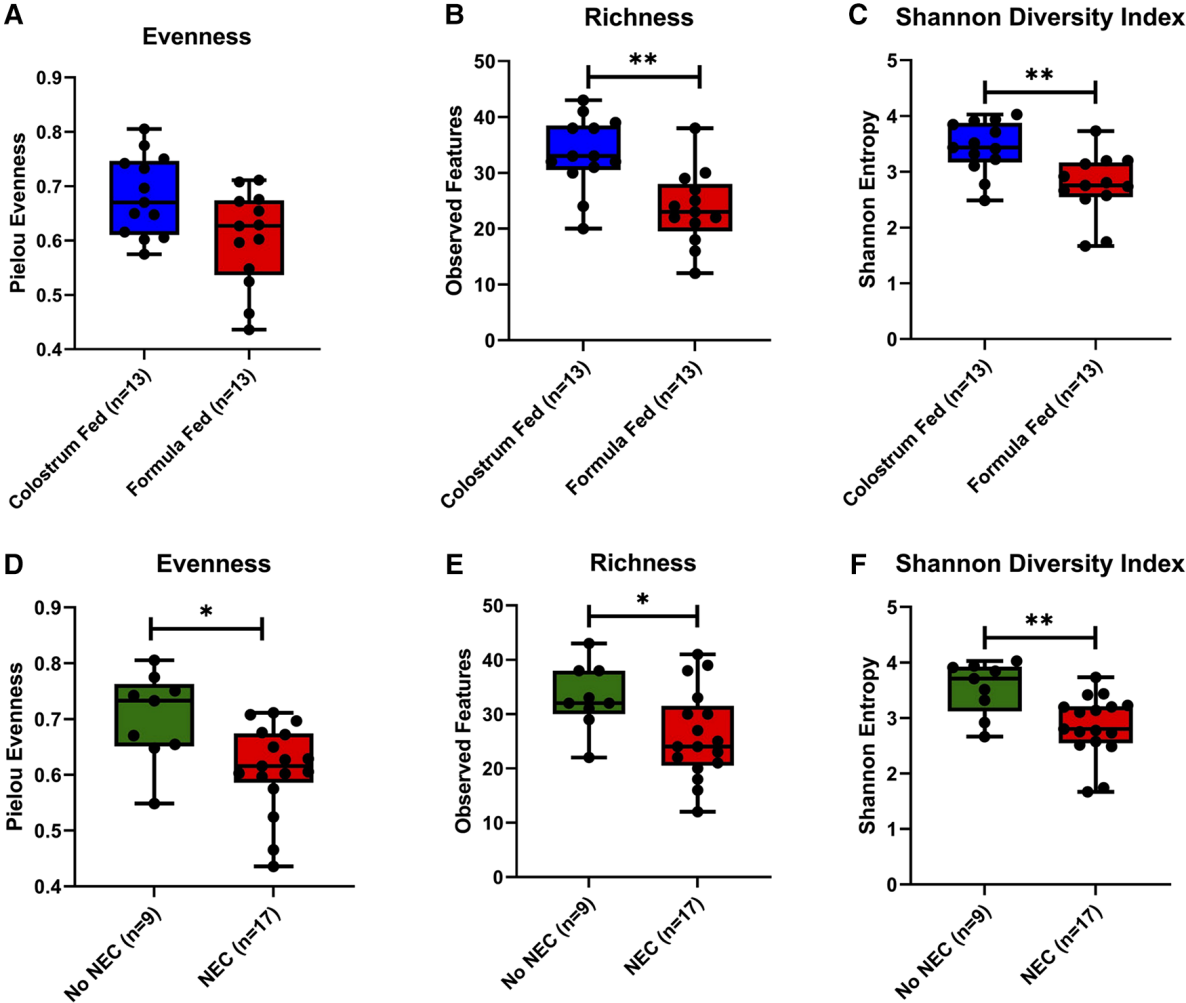
Microbial alpha diversity in piglet colonic contents. (**A,D**) Evenness within feeding regimen (*p *= 0.054) or occurrence of NEC (*p *= 0.012). (**B,E**) Richness (number of observed features) within feeding regimen (*p *= 0.002) or occurrence of NEC (*p *= 0.038). (**C,F**) Shannon Diversity Index within feeding regimen (*p *= 0.003) or occurrence of NEC (*p *= 0.006). Pairwise comparisons between groups were made using the Kruskal-Wallis test (*p *< 0.05). Data are represented as box and whisker plots that denote minimum, maximum, and interquartile range values.

To determine the influence of the feeding regimen and the impact of NEC on colonic microbial diversity within samples, alpha diversity was assessed based on evenness, richness, and Shannon Diversity Index, which accounts for both evenness and richness. Formula-fed piglets had a lower mean evenness score than colostrum-fed piglets (*p *= 0.054) (Figure 5A), as well as a significant decrease in richness (*p *= 0.002) (Figure 5B). Consistent with the observations of evenness and richness, the overall diversity within samples as denoted by the Shannon Diversity Index was significantly lower in the formula-fed relative to the colostrum-fed piglets (*p *= 0.003) (Figure 5C). When collapsed across feeding types, piglets that developed NEC during the experimental period had significantly reduced evenness (*p *= 0.012) and richness (*p *= 0.038) within colonic microbial communities relative to piglets that did not develop NEC (Figures 5D,E). Similarly, the Shannon Diversity Index was significantly reduced in piglets that developed NEC (*p *= 0.006) (Figure 5F). Analogous significant results were obtained when other alpha diversity measures were evaluated, including ACE, and Simpson Diversity Indices, but not Faith Phylogenetic Diversity (Supplementary Figure S3).

**Figure 6:**
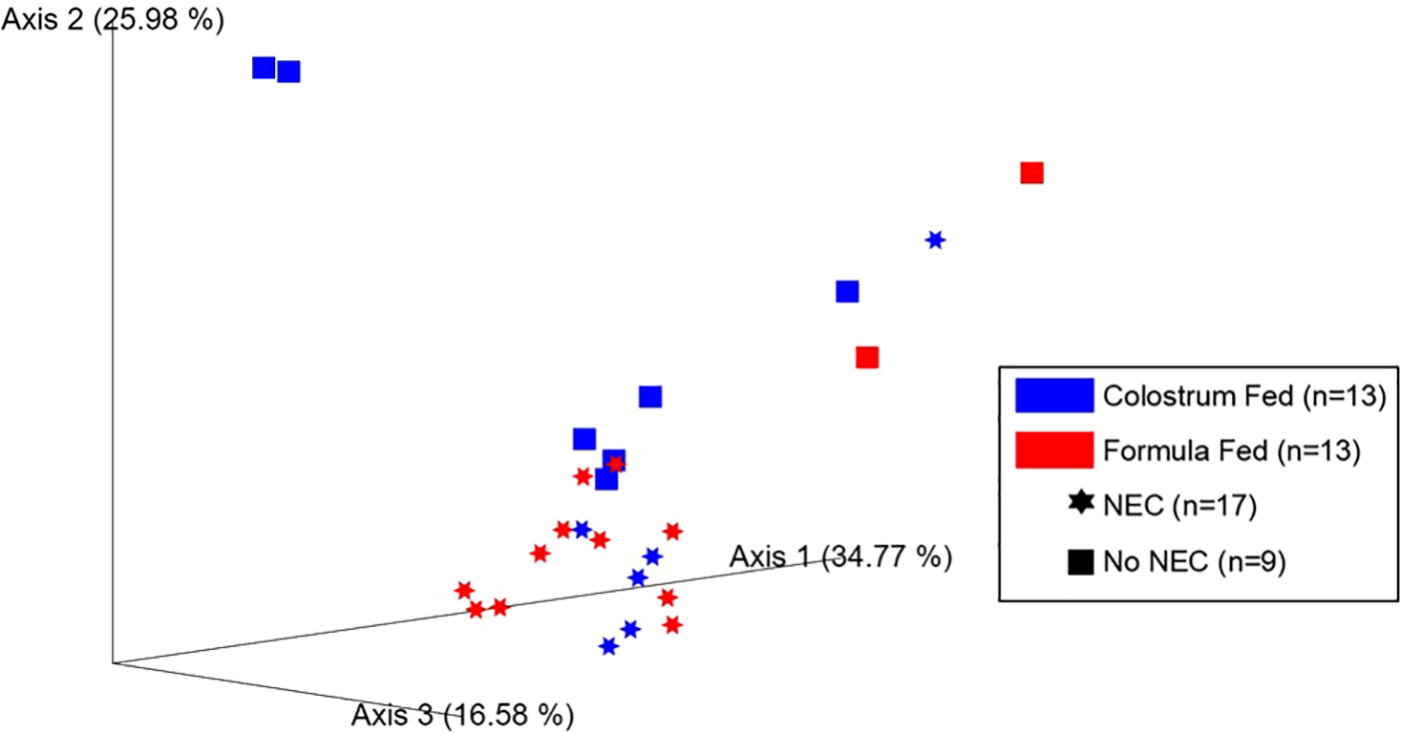
Microbial beta diversity in piglet colonic contents. Principal coordinate analysis (PCoA) plot of beta diversity clustering based on feeding regimen (*p *= 0.019, PERMANOVA using 999 randomizations of the data) and occurrence of NEC (*p *= 0.002, PERMANOVA using 999 randomizations of the data) derived from the weighted UniFrac distance matrix.

The weighted UniFrac distance matrix was constructed to characterize changes in the community and phylogenetic composition. Both feeding regimens (*p *= 0.019) and NEC (*p *= 0.002) were associated with significant changes in colonic content beta diversity. Importantly, a notable amount of clustering was associated with a positive NEC diagnosis (Figure 6). Thus, feeding regimen and NEC exerted independent influences on the bacterial composition within the colonic environment resulting in phylogenetically distinct microbial profiles. Comparable results were observed upon assessment of additional beta diversity metrics, including Bray-Curtis dissimilarity and Jaccard distances, but no differences were observed for the unweighted UniFrac distance matrix (Supplementary Figure S4).

### 3.5. NEC in piglets is associated with a significant change in microbial communities in the intestine

The relative abundances of specific bacterial taxa (at the class, family, and genus levels) were compared between the formula-fed and the colostrum-fed piglets, and between piglets that had or had not developed NEC (collapsed across the two feeding regimens). Taxa relative abundance bar plots were also generated at each of the previously described phylogenetic levels on an individual sample basis for feeding regimen (Supplementary Figures S5A–C) and occurrence of NEC (Supplementary Figures S6A–C).

**Figure 7:**
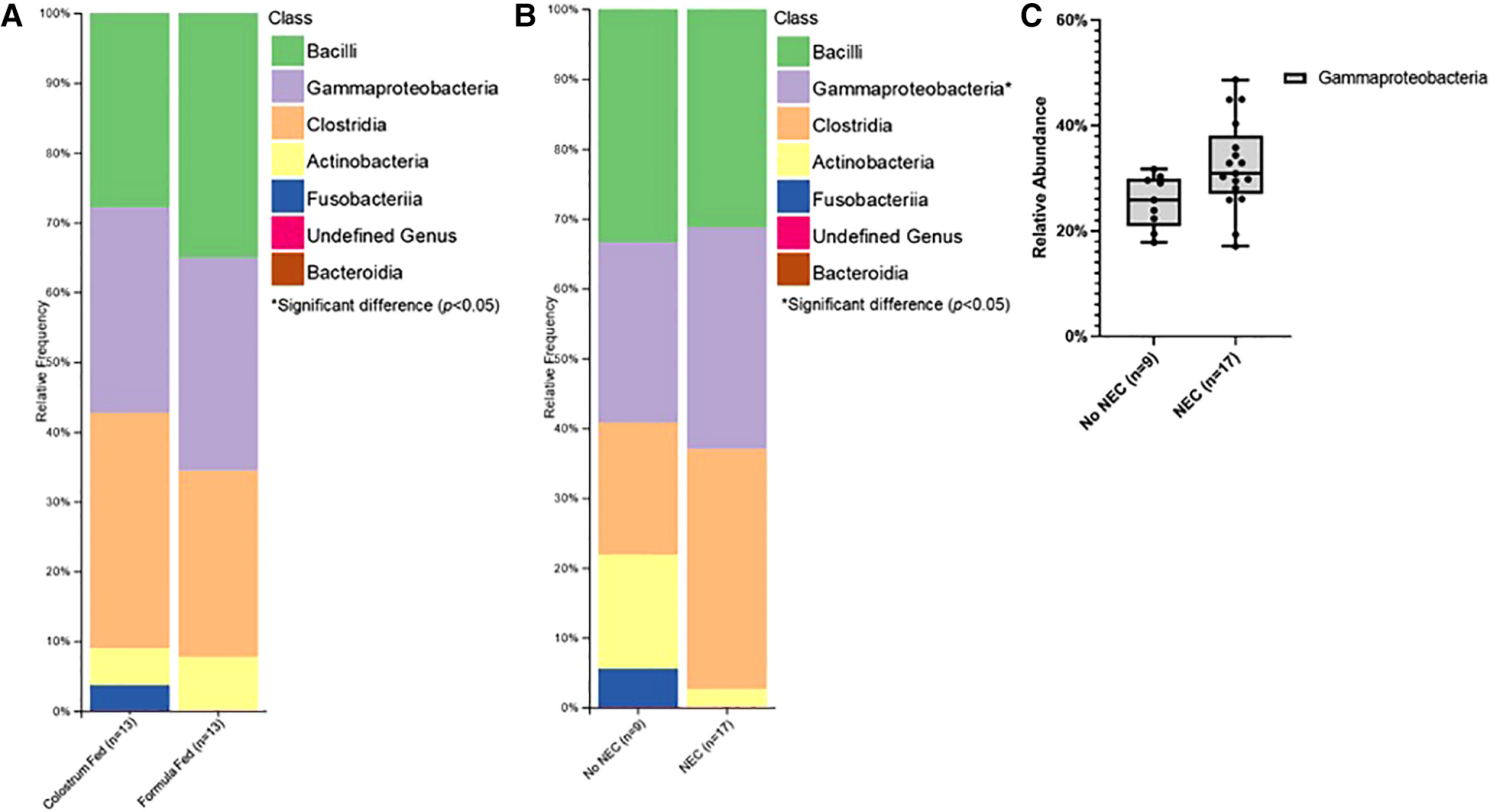
Class level taxonomic abundance. Class level taxa distribution within (**A**) feeding regimen and (**B**) occurrence of NEC. Bars represent mean relative percentage of each corresponding bacterial class. (**C**) Distribution of significantly different classes within incidence of NEC as box and whisker plots that denote minimum, maximum, and interquartile range values. For all relative abundance data, pairwise comparisons between groups were made using the Kruskal-Wallis test (*p *< 0.05).

**Figure 8:**
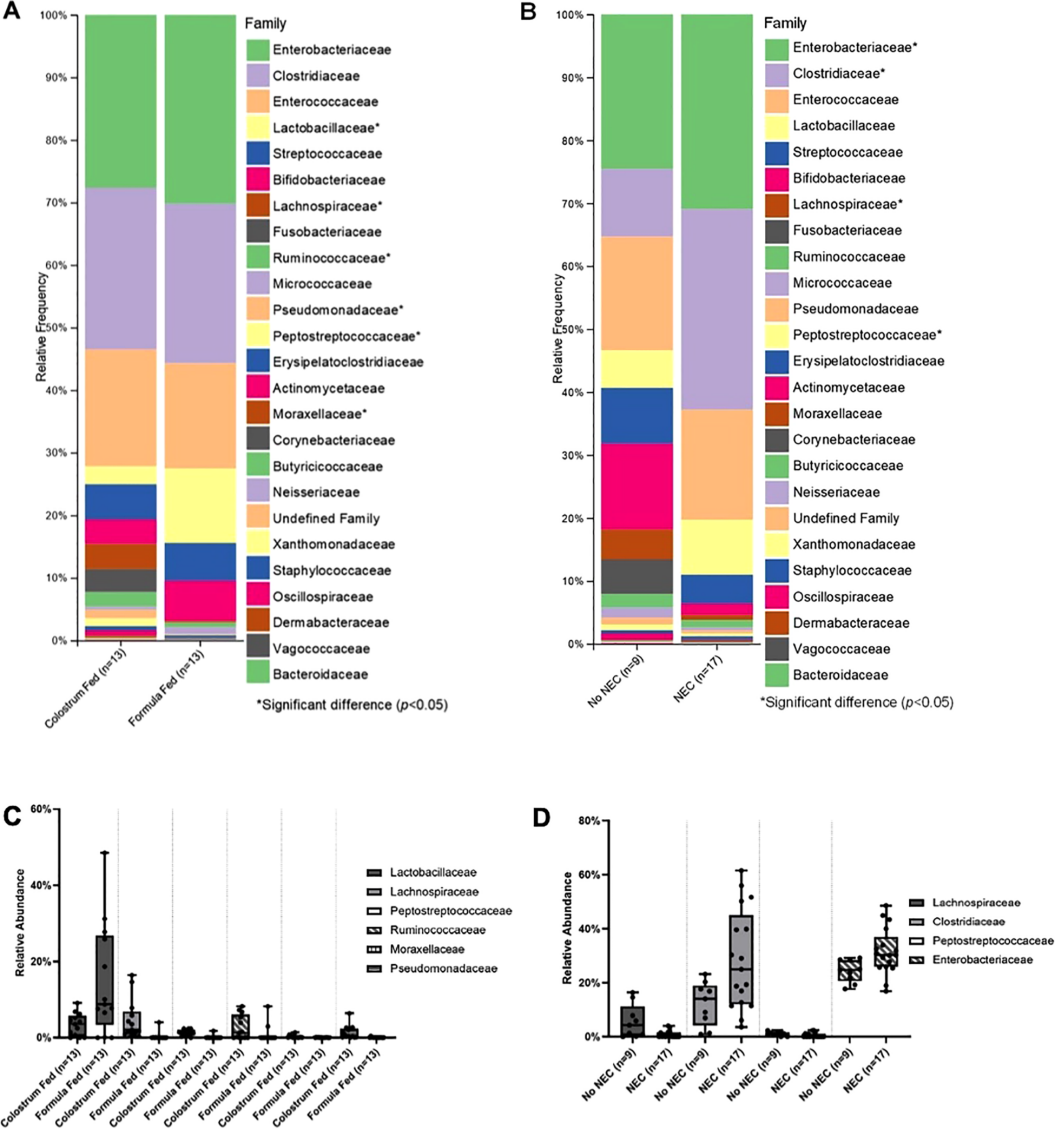
Family level taxonomic abundance. Family level taxa distribution within (**A**) feeding regimen and (**B**) occurrence of NEC. Bars represent mean relative percentage of each corresponding bacterial family. Distribution of significantly different families within (**C**) feeding regimen and (**D**) occurrence of NEC as box and whisker plots that denote minimum, maximum, and interquartile range values. For all relative abundance data, pairwise comparisons between groups were made using the Kruskal-Wallis test (*p *< 0.05).

**Figure 9:**
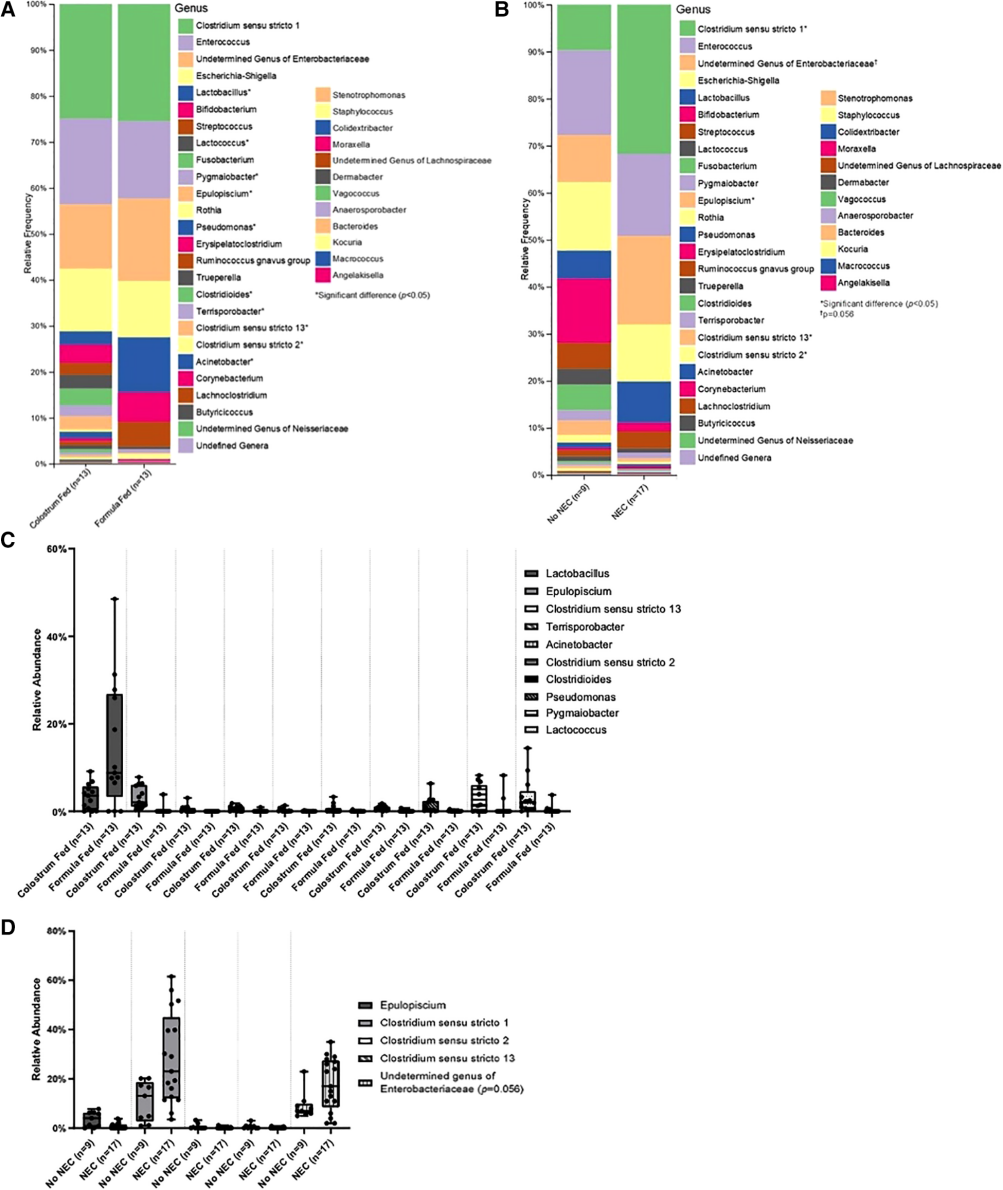
Genus level taxonomic abundance. Genus level taxa distribution within (**A**) feeding regimen and (**B**) occurrence of NEC. Bars represent mean relative percentage of each corresponding bacterial genera. Distribution of significantly different genera within (**C**) feeding regimen and (**D**) occurrence of NEC as box and whisker plots that denote minimum, maximum, and interquartile range values. For all relative abundance data, pairwise comparisons between groups were made using the Kruskal-Wallis test (*p *< 0.05).

Class level taxa were primarily dominated by Bacilli, Gammaproteobacteria, and Clostridia (Figures 7A,B). While there were no differences in the relative abundances of bacterial classes based on feeding regimen, there was a significant increase in the relative abundance of Gammaproteobacteria in piglets with NEC relative to piglets without NEC (*p *= 0.038) (Figure 7C). Enterobacteriaceae, Clostridiaceae, and Enterococcaceae predominated at the family classification level (Figures 8A,B). However, significant changes in the relative abundances of bacterial families occurred based on the two experimental factors (Figures 8C,D). The relative abundance of Lactobacillaceae was surprisingly higher in formula fed piglets than in colostrum fed piglets (*p *= 0.017). In contrast, colostrum-fed piglets had significantly increased relative abundance of Lachnospiraceae (*p *< 0.001), Peptostreptococcaceae (*p *< 0.001), Ruminococcaceae (*p *= 0.030), Moraxellaceae (*p *= 0.040) and Pseudomonadaceae (*p *= 0.001), (Figure 8C). Further, Lachnospiraceae (*p *= 0.013) and Peptostreptococcaceae (*p *= 0.047) were significantly more abundant in piglets without NEC. However, in piglets that developed NEC, Clostridiaceae (*p *= 0.029), and, importantly, Enterobacteriaceae (*p *= 0.01) abundances were significantly elevated relative to healthy piglets (Figure 8D). Similar to family level taxonomy, the most prevalent genera were similar when assessing based on feeding regimen or occurrence of NEC (Figures 9A,B). However, the relative abundances of bacterial genera differed between the two factors (Figures 9C,D). While ten genera were significantly different based on feeding (Figure 9C), the most notable was *Lactobacillus* (*p *= 0.017), which was elevated in formula-fed piglets, thus recapitulating family findings. *Epulopiscium* (*p *< 0.001), *Pseudomonas* (*p *= 0.001), *Pygmaiobacter* (*p *= 0.030), and *Lactococcus* (*p *= 0.001) were increased in colostrum-fed piglets (Figure 9C). *Epulopiscium* was also significantly higher in piglets without NEC (*p *= 0.013) (Figure 9D). In piglets that were positive for NEC, there was a significant increase in the abundance of *Clostridium sensu stricto* 1 (*p *= 0.025), *Clostridium sensu stricto* 2 (*p *= 0.021) and *Clostridium sensu stricto* 13 (*p *= 0.017) (Figure 9D). Additionally, an undefined genus of *Enterobacteriaceae* was increased in NEC-positive piglets with a trend towards significance (*p *= 0.056). Collectively, taxonomic abundance data indicate that colostrum and formula feeding differentially influence pioneer colonization within the colonic environment. Additionally, this pioneer colonization may, to an extent, also affect the distribution and prevalence of taxa seen here to be associated with the onset of NEC.

## 4. Discussion

Given the complexity of NEC pathophysiology, the development of animal models emulating different facets of the human disease is a critical step towards better disease understanding, and a prerequisite to efficient preclinical drug testing. Here, we describe a new model of NEC using premature piglets that are exclusively orally fed, eliminating the need for TPN, and that utilizes BC during the initial 24 h. Our preliminary studies revealed that exclusive formula feeding of premature piglets resulted in rapid mortality within the first 24 h of life, limiting the possibility of testing novel therapeutic strategies in laboratory settings. Given the importance of maternal antibodies in the protection against NEC, we then used BC to confer transient protection during the first hours of life. The subsequent transition from colostrum to formula resulted in the development of intestinal injury that is similar to human NEC. However, the protection conferred by BC allows for improved early survival and the use of our model in testing potential novel therapeutics. Unlike the commonly used rodent models, where a combination of TLR4 activation and hypoxic stress are required for the induction of the disease, NEC occurs spontaneously with formula feeding in the piglet model, making it more physiologically relevant. Interestingly, despite a significant reduction in NEC incidence with colostrum-only feeding, some animals in this group still developed the disease. It is possible that this is partially due to the use of BC as opposed to porcine colostrum. Although it does not typically occur this quickly, this is also observed with humans, where despite a substantial decrease in NEC incidence observed with exclusive breastfeeding, some infants can still develop the disease, likely due to etiologies that are still obscure (10, 11).

According to Center for Disease Control (CDC) data from 2017, the prevalence of infants receiving any breast milk in 48 states and the District of Columbia was 83.9% overall and varied by gestational age (53). Looking at specific age groups, 71.3% of extremely preterm infants (20–27 weeks), 76.0% of early preterm infants (28–33 weeks), 77.3% of late preterm infants (34–36 weeks), and 84.6% of term infants (≥37 weeks) received breast milk (53). Many preterm infants struggle to gain weight appropriately after birth due to immaturity of the gastrointestinal tract, feeding difficulties (i.e., immature sucking movements, low breastmilk supply), and a lack of appropriate hepatic glycogen stores (54, 55). Thus, to ensure that these infants receive adequate nutrition during this time, TPN is administered *via* a central venous catheter. Despite the high percentage of preterm infants receiving any breast milk, the incidence of proven or severe NEC in the United States is estimated to be approximately 1–3 per 1,000 live births (56). Unfortunately, NEC frequently occurs in susceptible preterm infants following the administration of enteral feeds. Studies conducted in premature piglets suggest that maltodextrin in human infant formulas is partly responsible for intestinal injury during NEC (57, 58). Thus, initial TPN administration followed by initiation of enteral feeds has been used in several established models of NEC in swine (16, 18, 24, 25, 31, 59, 60). Often, these studies continue the administration of TPN at lower rates while simultaneously administering enteral feeds for 18–120 h (18, 29–31, 37, 59–63). On the other hand, other investigators have developed piglet NEC models using enteral feeds exclusively for NEC induction (64–66). Good et al. used cultured enteric bacteria isolated from a patient with surgical NEC to induce intestinal lesions in neonatal mice and premature piglets (65). Similarly, Roy et al. directly administered fermented formula through a catheter inserted through a gastrostomy directly into the duodenum resulting in NEC-like disease within 6 h in piglets (66). Regardless of the model, the initiation of enteral feeds seems to trigger NEC, leading to rapid fatalities with as high as a 70% mortality rate within 48 h following the initial administration of formula (18, 24–26, 31). The severity of the disease and the subsequent rapid mortality in these models drastically limit the window of therapeutic intervention in the context of investigational studies aiming to assess the efficacy of novel treatments, making our model a potentially better option. In addition, while breast milk is known to be protective against NEC due to the presence of maternal antibodies, there are still some infants that exclusively receive breast milk and still develop NEC. Our model mimics these conditions, as we still observed some NEC in piglets in the colostrum-fed group, despite receiving beneficial antibodies from colostrum administration.

Traditionally, the severity of NEC in piglets has been assessed using gross injury scoring which depicts macroscopic damage of the intestine, or histologic injury scoring, or a combination of both (18, 24–26, 29–31, 37, 59, 61–63, 65, 68). During our study, it was common to find piglets with high gross injury and high histologic injury scores that appeared clinically healthy throughout the entire experiment and did not show bacterial translocation to internal organs. Moreover, the histologic injury score did not reflect the extent of damage throughout the length of the intestine, as the tissue specimens collected for histological assessment, as in previous studies using piglet NEC models, were obtained from the areas of the intestine displaying the most severe macroscopic lesions (24–26, 31, 64, 65, 68). Therefore, we developed a new D-NEC score that entailed a clinical sickness scoring system and assessment of bacterial translocation (5, 67). This scoring system also reduces the likelihood of attributing death during the experiment to other potential etiologies common in premature piglets, such as lung immaturity, inadequate thermoregulation, and sepsis (16). A similar approach using a multi-component scoring system that included gross injury, radiographic findings on abdominal x-ray after euthanasia, and pre-mortem clinical signs of NEC was used by Azcarate-Peril et al*.* (64). A multi-component scoring system is also reminiscent of that first described by Bell and used by Neonatologists to diagnose patients with NEC (4). In agreement with previous reports, our new scoring system was further strengthened by the quantification of inflammatory factors which revealed a significant upregulation of *IL-1α* and *IL-10* in the colon of animals that developed NEC (68–76).

Despite multiple gaps in our understanding of NEC etiology and pathophysiology, the contribution of the microbiome to the development of NEC, initially suggested by Claud and Walker (77), has been widely documented in the last decade (78–82). Several studies have clearly established that intestinal bacterial diversity in premature infants is inherent to gestational age, breastfeeding, and the use of antibiotics (83, 84). A decrease in microbial diversity is often associated with the onset of NEC (77, 81, 85–87). Our data revealed that prolonged formula feeding decreases bacterial diversity, similar to the findings in premature human infants (88). Furthermore, we found that NEC, whether in the colostrum-fed or formula-fed group, was associated with low microbial diversity. Interestingly, we also showed that the development of NEC in our model is associated with increased Gammaproteobacteria and Enterobacteriaceae. This observation is in line with previous reports showing that NEC is consistently associated with the enrichment of Proteobacteria and Enterobacteriaceae in preterm infant cohorts (77, 85, 88, 89). Findings that Gram-negative bacteria dominate the gut microbiota of preterm infants are consistent with the hypothesis that increased Proteobacteria is directly linked to the development of NEC through the activation of TLR4 (11, 14, 90, 91). Indeed, it is widely accepted that the premature intestine is more prone to TLR4-driven inflammation, which is likely a primary cause of NEC (11, 14, 90, 91). Our data also revealed that NEC was associated with a significant increase in Clostridiaceae abundance (64, 87, 92). Despite the absence of a consensus regarding the association with NEC, a few studies have similarly shown an increase of Clostridiaceae in NEC patients (85, 86, 89). We hypothesize that formula-feeding in our model more strongly drives microbial colonization profiles associated with NEC, and it is this altered microbial colonization that contributes TLR-4 driven inflammation. While our model displayed similarities with microbial changes observed in NEC patients, it also showed some differences. In contrast with the significant decrease of Firmicutes described in premature babies diagnosed with NEC (9), our piglets that developed NEC had a significantly higher relative abundance of Firmicutes than the control animals (data not shown). It is worth noting that it is challenging to identify the microbiome changes that are exclusively associated with human NEC, since administering antibiotics to premature infants is a broadly used practice in neonatal intensive care units. Therefore, antibiotic therapy cannot be excluded as a causative factor of some of the changes observed in microbiome analyses in NEC patients (93). It is also possible that the differences observed between human and swine NEC are specific to each species.

Our model relies on the transitional protection conferred by colostrum prior to formula feeding. Although at a much lower incidence, injury in the colostrum-fed group is still observed. This possibly occurs because we do not use TPN for supplemental nutrition to allow for delayed introduction of enterally-administered colostrum. As a control group, this allows us to discern the baseline incidence of NEC in piglets receiving enteral colostrum only, similar to infants receiving enteral breast milk only. Given the batch-to-batch differences of antibody titers in colostrum, it is imperative to assess the quality of the colostrum prior to use. This variability in the concentration of antibodies in breast milk is also observed in humans (94). Furthermore, we cannot definitively ascertain the nutritional and caloric content of BC, as can be done with Neocate Junior infant formula.

In conclusion, we have developed a new enteral feed-only model of NEC in premature piglets with an initial period of BC administration to improve survival and have introduced a novel multifactorial D-NEC scoring system. Similar to humans, premature piglets in this model spontaneously develop a NEC-like disease upon initiation of enteral formula feeds. NEC in this model is characterized by the impairment of the epithelial barrier leading to bacterial translocation and a deleterious inflammatory response. We have also demonstrated that our model recapitulates the main features of microbial dysbiosis seen in NEC patients. Moving forward, this model can be used for pre-clinical testing of novel therapies for NEC in order to facilitate the transition of these novel therapies from bench to bedside.

## Data Availability

The datasets presented in this study can be found in online repositories. The names of the repository/repositories and accession number(s) can be found here: 16S rRNA sequencing data is available in Sequence Read Archive (SRA) under BioProject accession number PRJNA922570.
