# Supplementary material for: Development of a novel definitive scoring system for an enteral feed-only model of necrotizing enterocolitis in piglets
Source: Front Pediatr. 2023 Apr 17;11:1126552. doi: 10.3389/fped.2023.1126552 (PMC10149862; doi:10.3389/fped.2023.1126552)
Supplement: Supplementary file 1 [file Datasheet1.docx]

Supplementary Material

Development of a Novel Definitive Necrotizing Enterocolitis (NEC) Scoring System for an Enteral Feed-Only Model of NEC in Piglets

**Mecklin V. Ragan^1,2^, Samantha J. Wala^1,2^, Nitin Sajankila^1,2^, Audrey F. Duff ^3^, Yijie Wang^1^, Samuel G. Volpe^1^, Ameer Al-Hadidi^1^, Zachary Dumbauld^1^, Nanditha Purayil^1^, Joseph Wickham^3^, Miriam R. Conces^4^, Belgacem Mihi^1^, Steven D. Goodman^3^, Michael T. Bailey^3^,
Gail E. Besner^*,1,2^**

^1^Center for Perinatal Research, Nationwide Children’s Hospital, Columbus, Ohio, United States

^2^Department of Pediatric Surgery, Nationwide Children’s Hospital, Columbus, Ohio, United States

^3^Center for Microbial Pathogenesis, Nationwide Children’s Hospital, Columbus, Ohio, United States

^4^Department of Pathology, Nationwide Children’s Hospital, Columbus, Ohio, United States

*** Correspondence:**Gail E. Besner, MD

Department of Surgery

Nationwide Children’s Hospital

700 Children’s Drive

Columbus, OH 43205

Phone: (614) 722-3914

[gail.besner@nationwidechildrens.org](mailto:gail.besner@nationwidechildrens.org)

## Supplementary Figure Legends

**Supplementary Figure 1. Pig NEC Model.** Premature piglets were delivered via Cesarean section on day of life (DOL) 0. All piglets received bovine colostrum for the first 24 h. Piglets in Group 1, the colostrum-fed group, received colostrum for the remainder of the experiment. Piglets in Group 2, the formula-fed group, began receiving Neocate Junior formula after the first 24 h. Piglets were sacrificed when they met euthanasia criteria or at the end of the experiment on day 5. At the time of necropsy, internal organs, intestinal samples and stool samples were collected for further processing.

**Supplementary Figure 2. Regional Intestinal Injury. (A)** Gross injury scores by region of intestine. **(B)** Histologic injury scores by region of intestine. Variability of sample means was reported as a standard error of the mean (SEM).

**Supplementary Figure 3. Additional Alpha Diversity Indices. (A)** ACE Index within feeding regimen (*p*=0.002) and incidence of NEC (*p*=0.038). **(B)** Chao1 Index within feeding regimen (*p*=0.002) and incidence of NEC (*p*=0.038). **(C)** Faith Phylogenetic Diversity Index within feeding regimen (*p*=0.397) and incidence of NEC (*p*=0.500). **(D)** Simpson Diversity Index within feeding regimen (*p*=0.011) and incidence of NEC (*p*=0.003). Pairwise comparisons between groups were made using the Kruskal-Wallis test (*p*<0.05). Data are represented as box and whisker plots that denote minimum, maximum, and interquartile range values.

**Supplementary Figure 4. Additional Alpha Diversity Indices. Principal coordinate analysis (PCoA) plots of beta diversity clustering. (A)** Effects of feeding regimen (*p*=0.087) and incidence of NEC (*p*=0.148) derived from the unweighted UniFrac distance matrix. **(B)** Effects of feeding regimen (*p*=0.001) and incidence of NEC (*p*=0.029) derived from Jaccard distance matrix. **(C)** Effects of feeding regimen (*p*=0.008) and incidence of NEC (*p*=0.010) derived from Bray-Curtis dissimilarity matrix. All matrix distances were analyzed by PERMANOVA using 999 randomizations of the data.

**Supplementary Figure 5.** **Taxonomic Relative Abundance Within Individual Samples Based on Treatment. (A)** Class level taxa distribution within feeding regimen. Bars represent mean relative percentage of each corresponding bacterial class. **(B)** Family level taxa distribution within feeding regimen. Bars represent mean relative percentage of each corresponding bacterial families. **(C)** Genus level taxa distribution within feeding regimen. Bars represent mean relative percentage of each corresponding bacterial genera. For all relative abundance data, pairwise comparisons between groups were made using the Kruskal-Wallis test (*p*<0.05).

**Supplementary Figure 6. Taxonomic Relative Abundance Within Individual Samples Based on Incidence of NEC. (A)** Class level taxa distribution within incidence of NEC. Bars represent mean relative percentage of each corresponding bacterial class **(B)** Family level taxa distribution within incidence of NEC. Bars represent mean relative percentage of each corresponding bacterial families. **(C)** Genus level taxa distribution within incidence of NEC. Bars represent mean relative percentage of each corresponding bacterial genera. For all relative abundance data, pairwise comparisons between groups were made using the Kruskal-Wallis test (*p*<0.05).
