# Supplementary figures and images for: Development of a novel definitive scoring system for an enteral feed-only model of necrotizing enterocolitis in piglets
Source: Front Pediatr. 2023 Apr 17;11:1126552. doi: 10.3389/fped.2023.1126552 (PMC10149862; doi:10.3389/fped.2023.1126552)

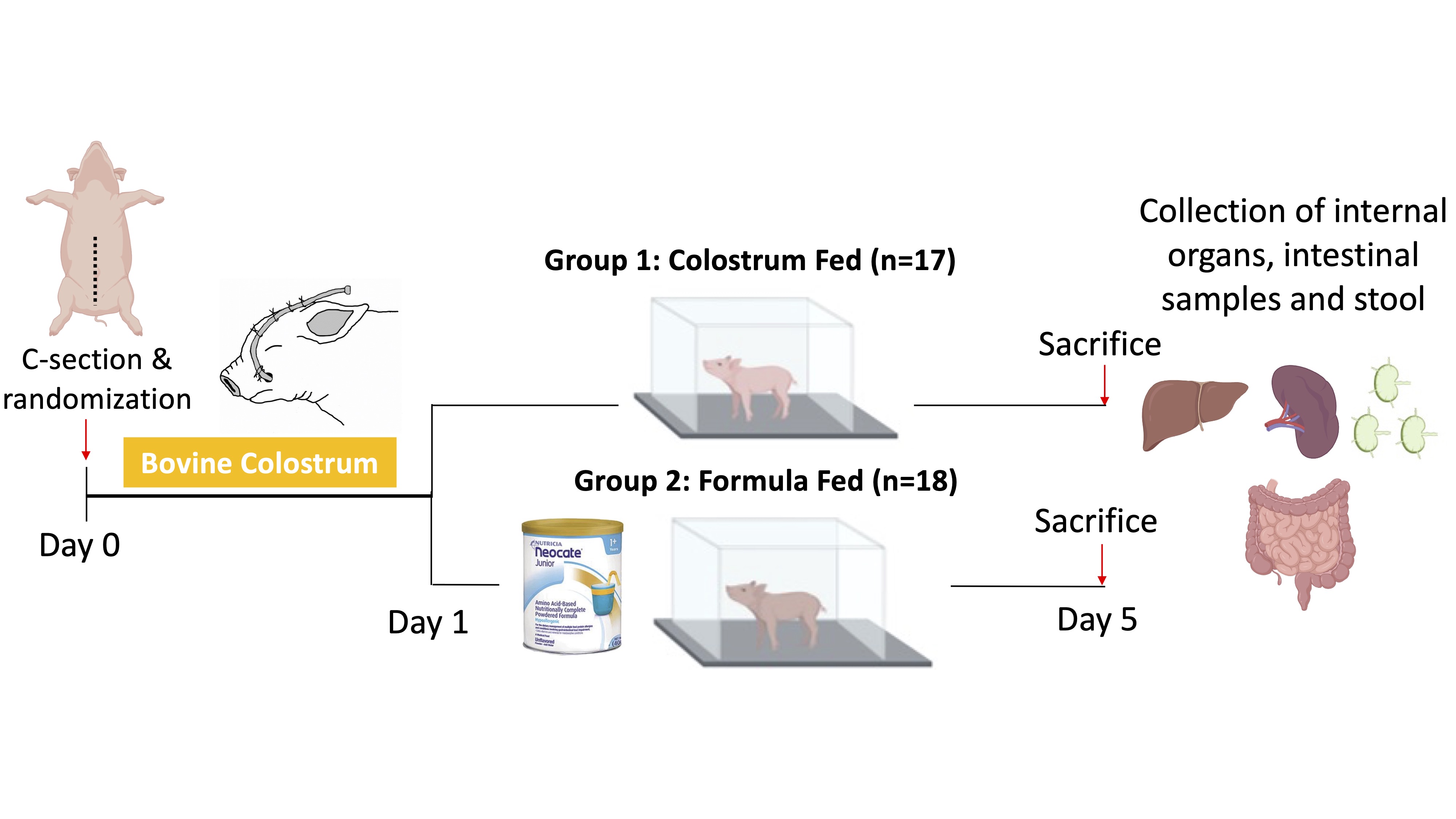

Supplement: Supplementary file 2 [file Image1.jpeg]

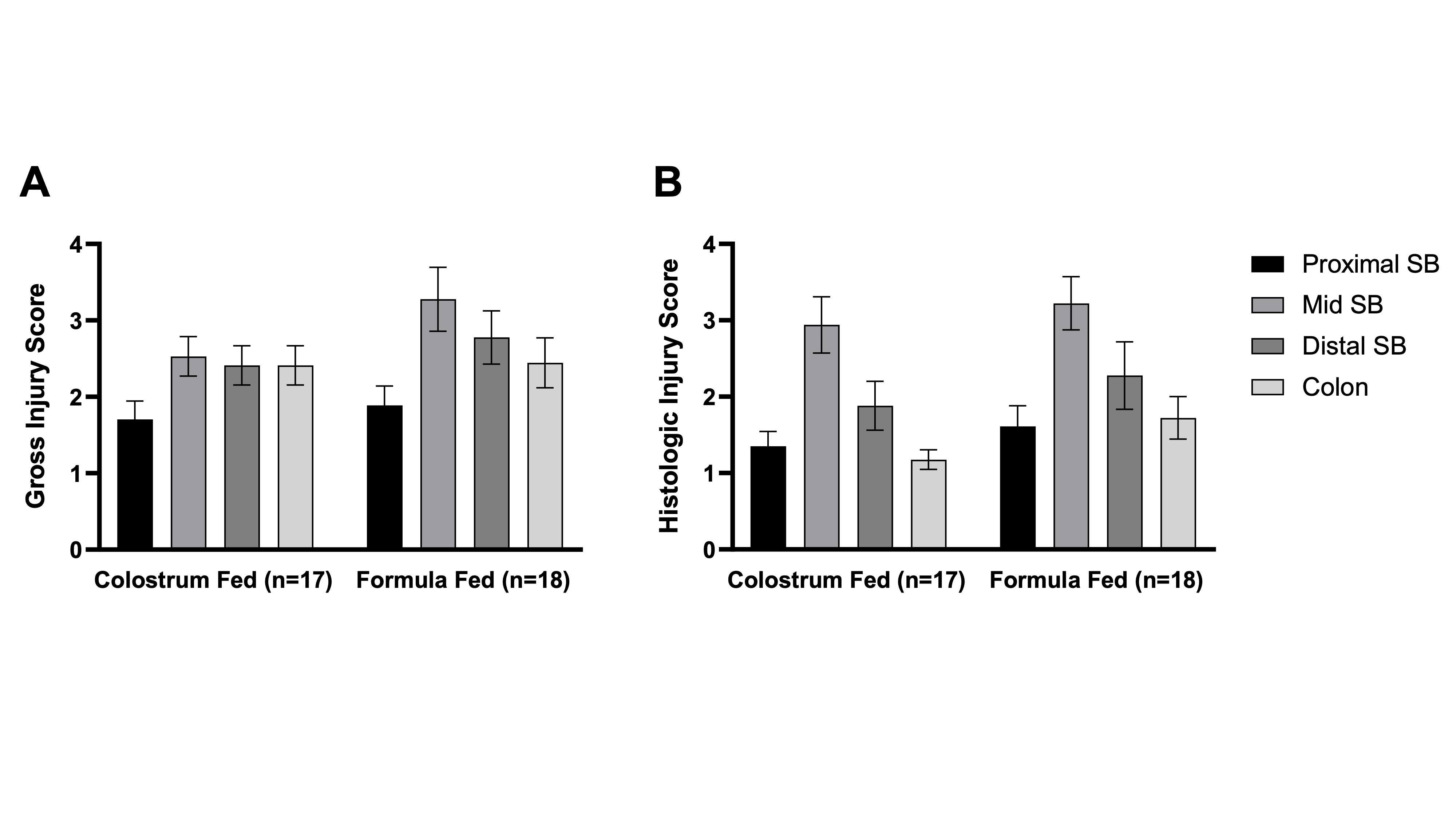

Supplement: Supplementary file 3 [file Image2.jpeg]

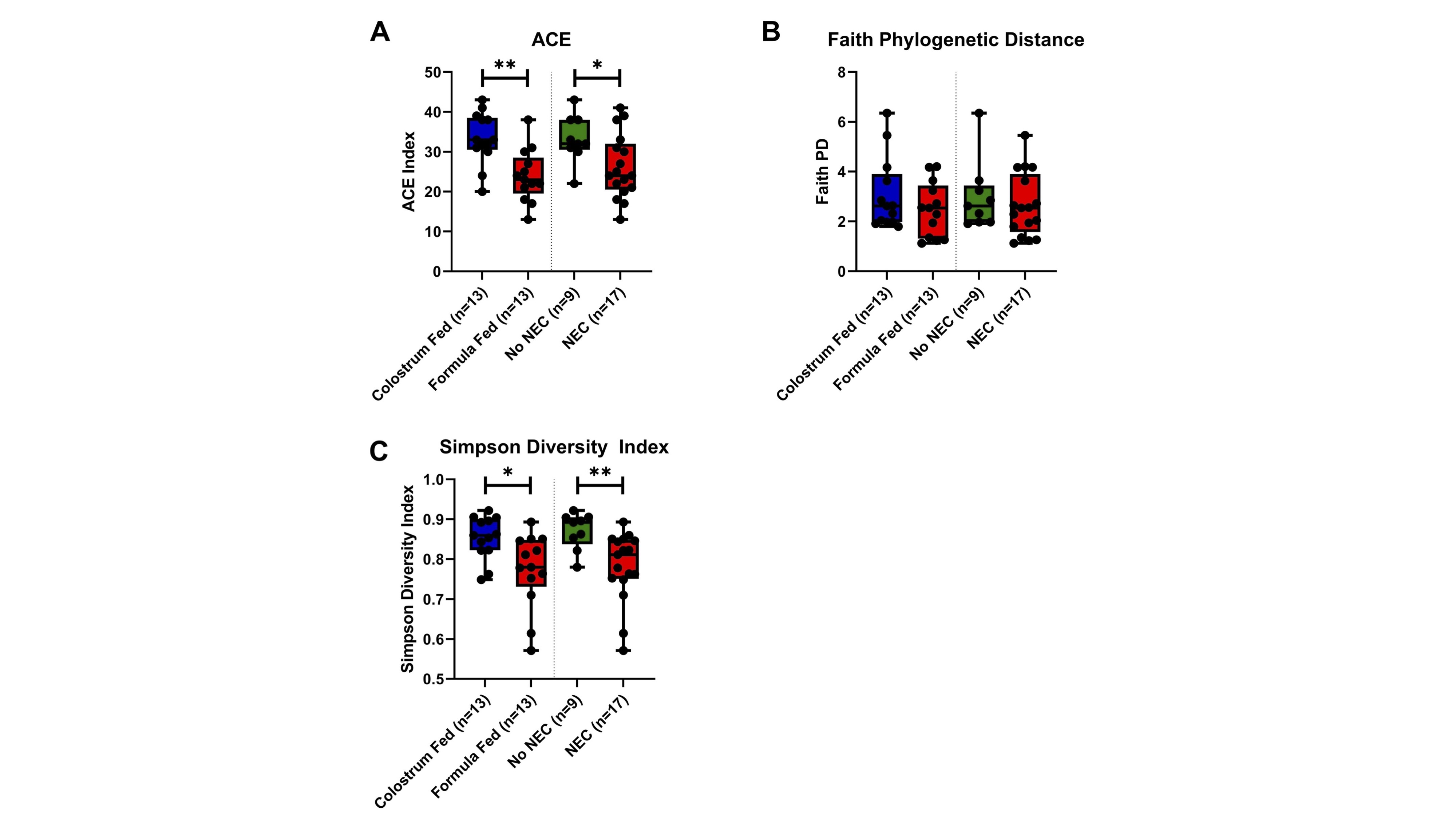

Supplement: Supplementary file 4 [file Image3.jpeg]

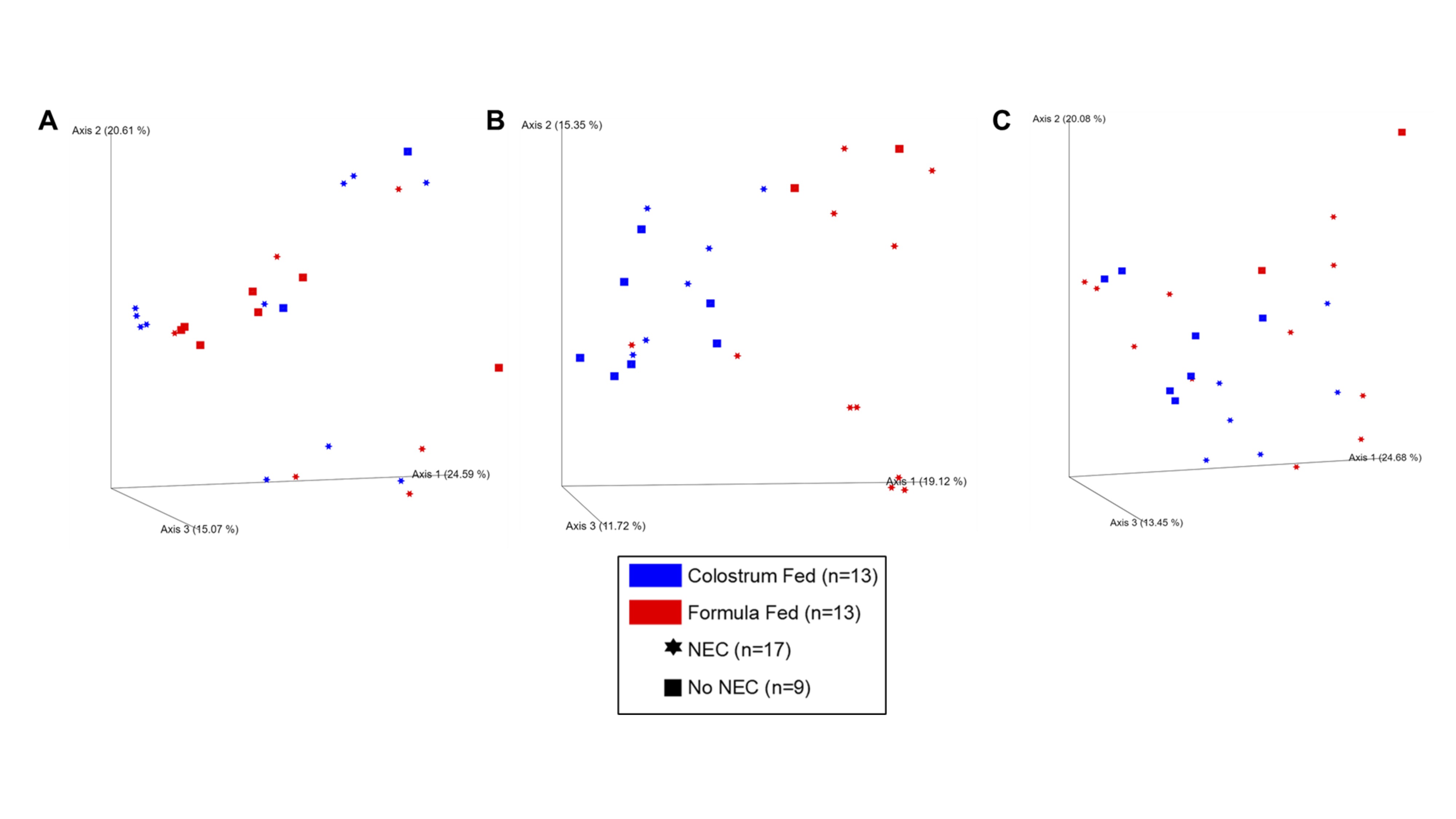

Supplement: Supplementary file 5 [file Image4.jpeg]

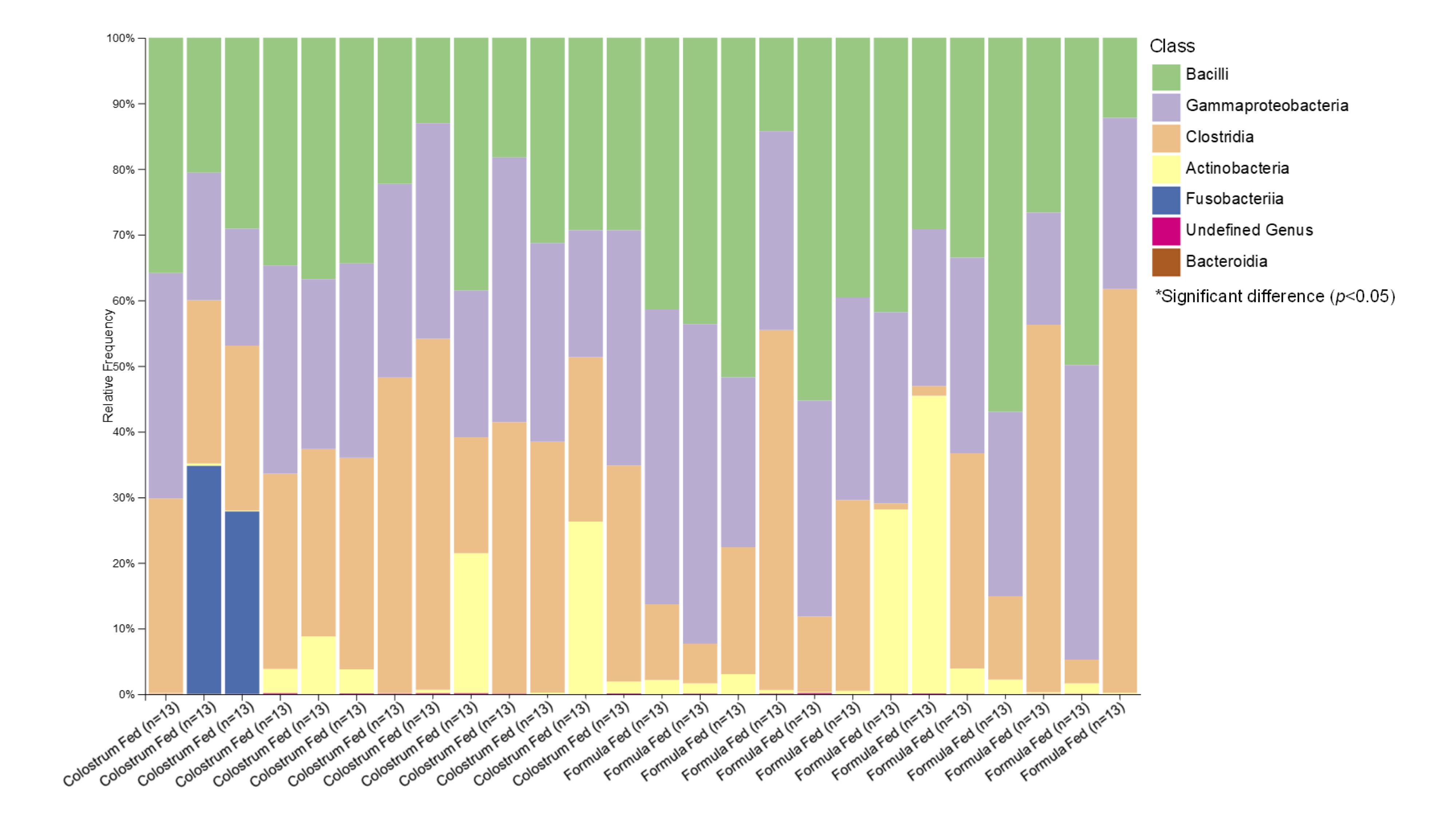

Supplement: Supplementary file 6 [file Image5.jpeg]

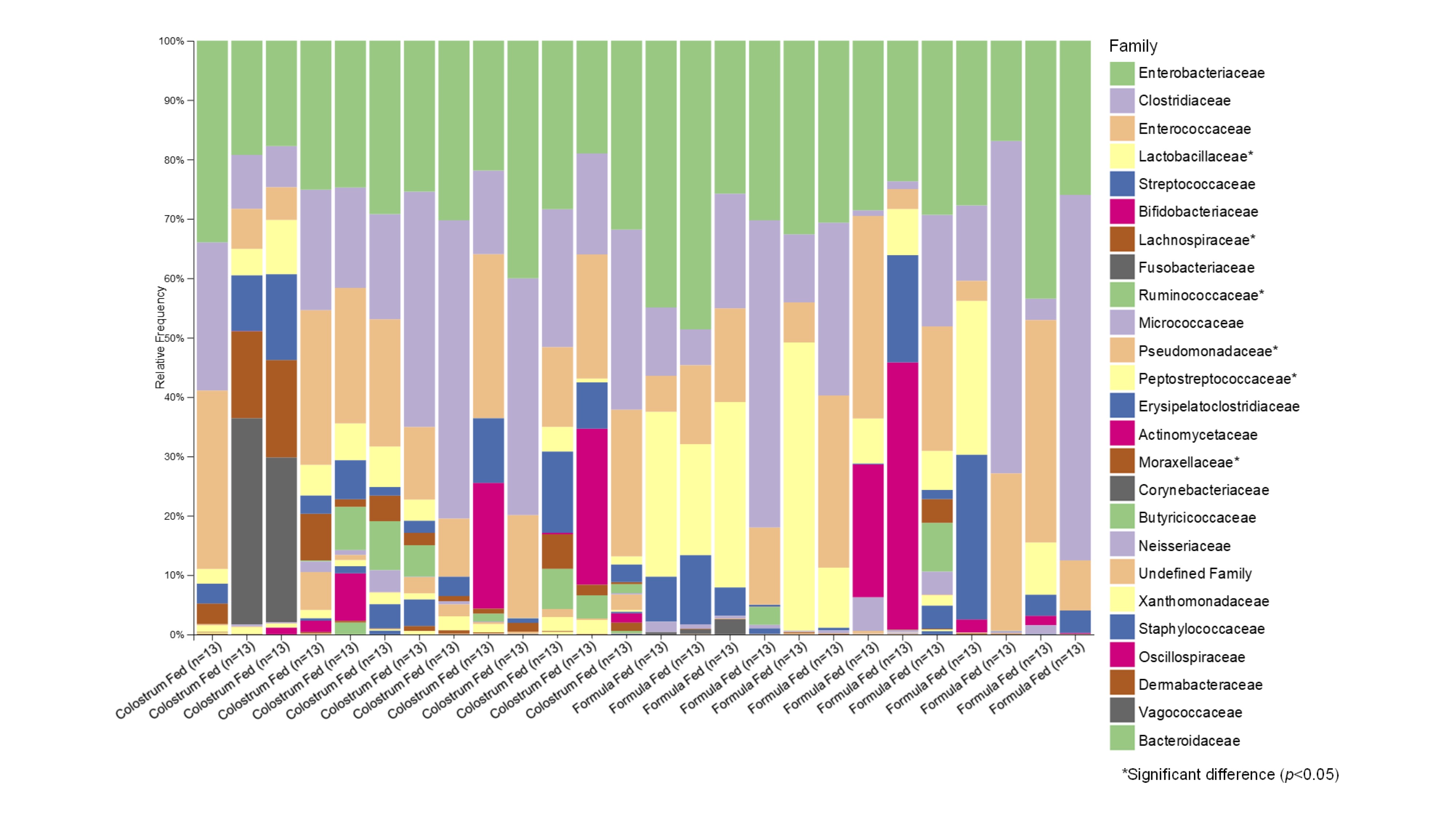

Supplement: Supplementary file 7 [file Image6.jpeg]

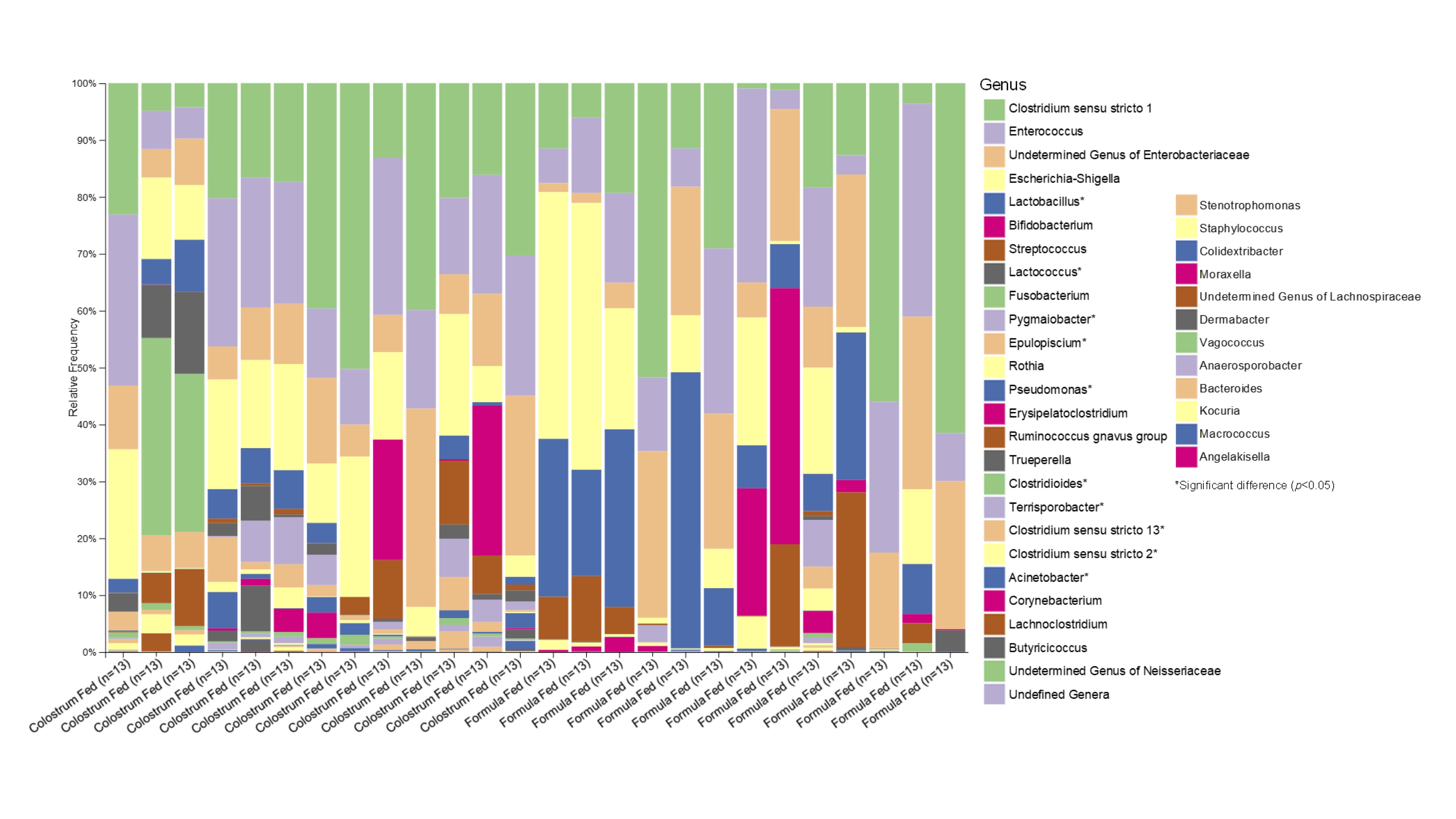

Supplement: Supplementary file 8 [file Image7.jpeg]

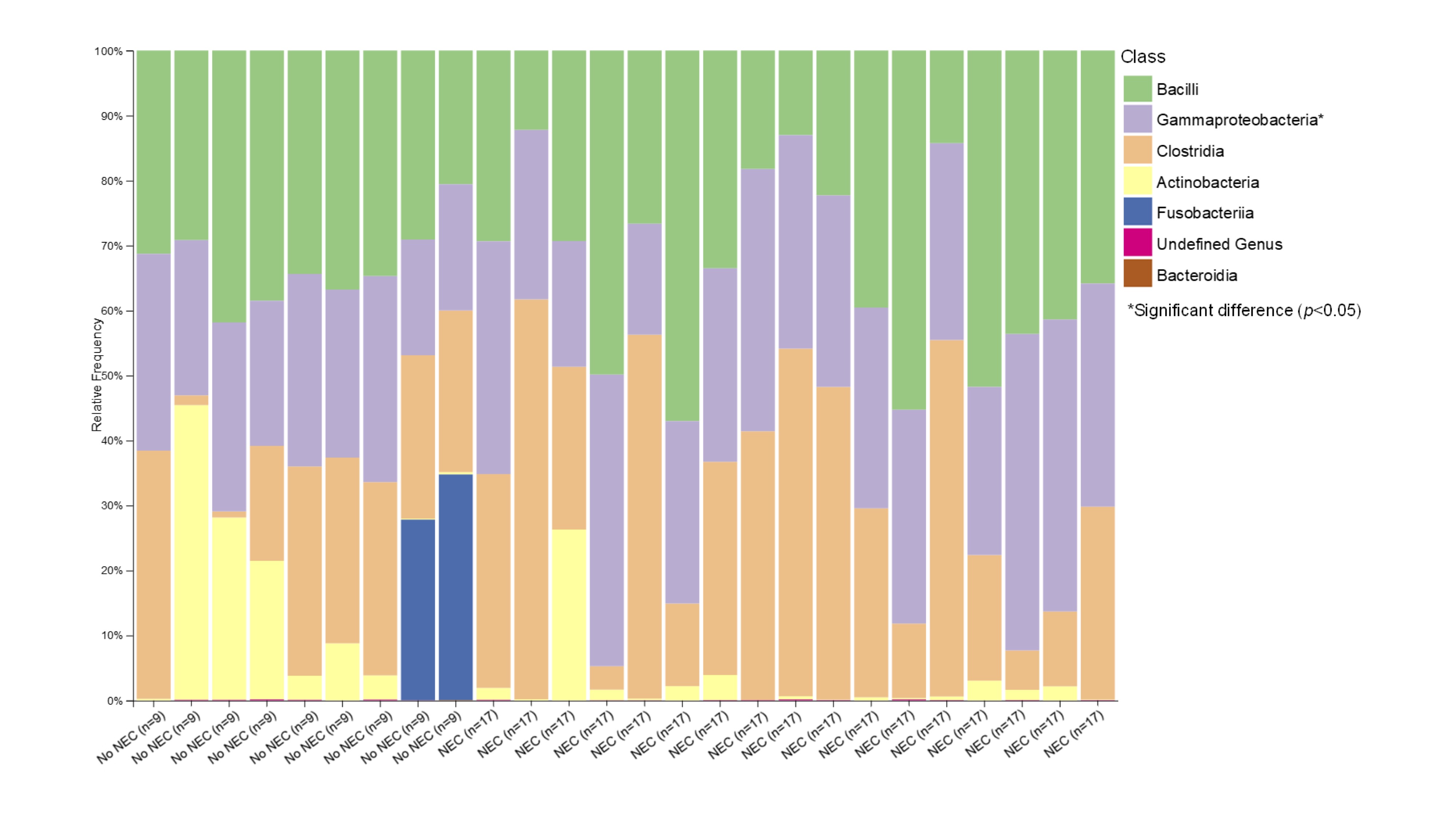

Supplement: Supplementary file 9 [file Image8.jpeg]

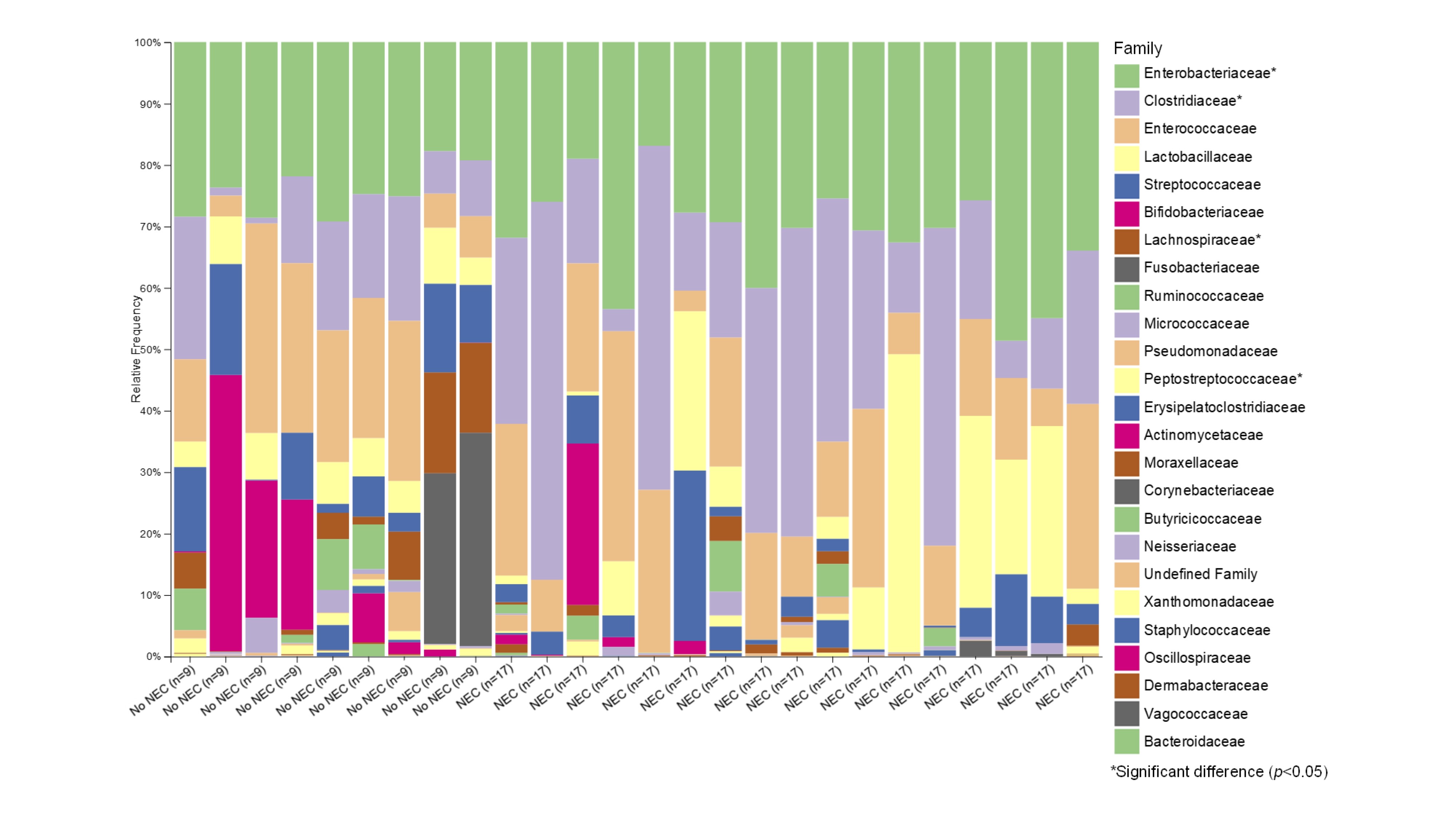

Supplement: Supplementary file 10 [file Image9.jpeg]

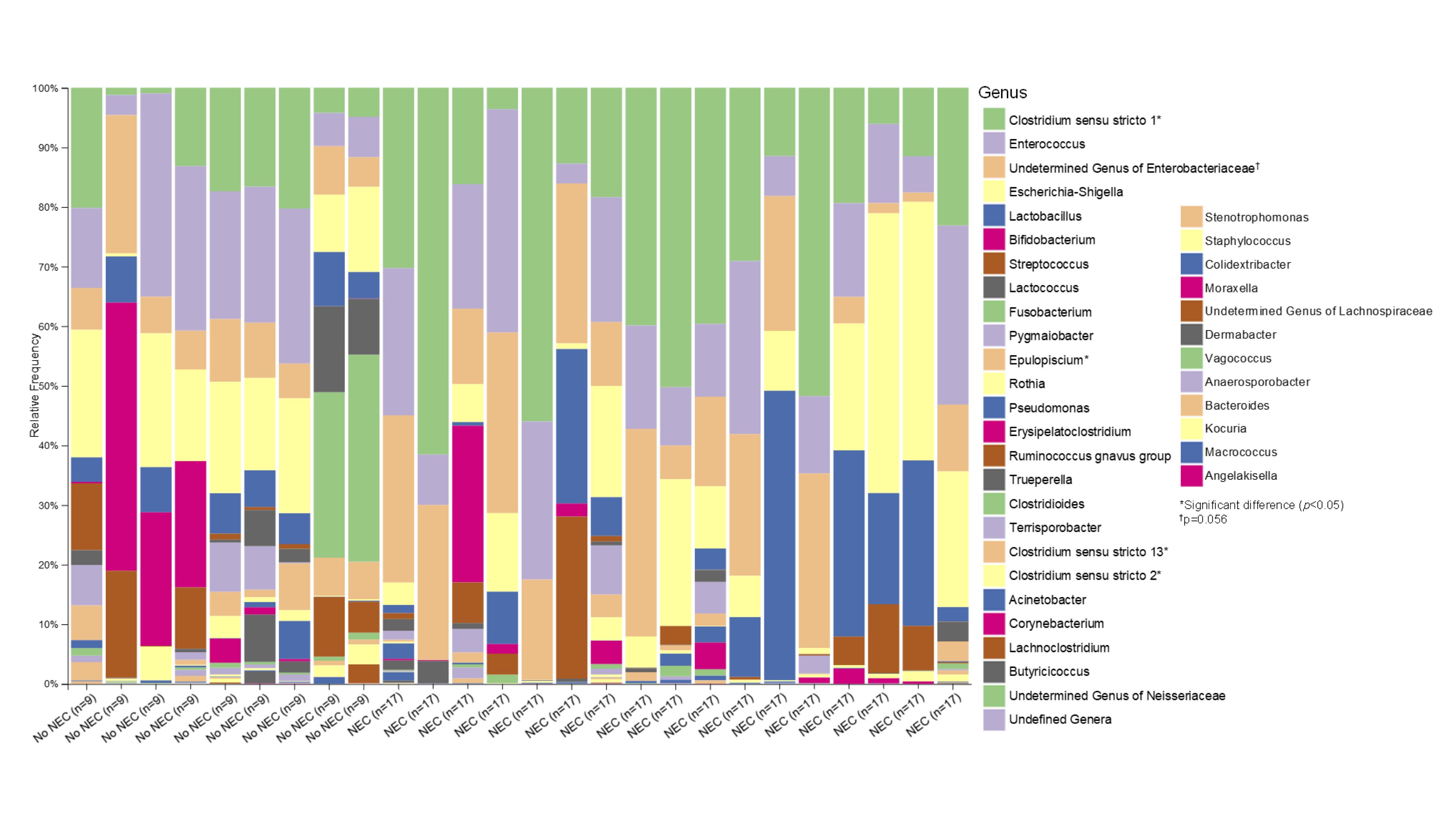

Supplement: Supplementary file 11 [file Image10.jpeg]
